# Trait variation and genetic diversity in a banana genomic selection training population

**DOI:** 10.1371/journal.pone.0178734

**Published:** 2017-06-06

**Authors:** Moses Nyine, Brigitte Uwimana, Rony Swennen, Michael Batte, Allan Brown, Pavla Christelová, Eva Hřibová, Jim Lorenzen, Jaroslav Doležel

**Affiliations:** 1 Faculty of Science, Palacký University, Olomouc, Czech Republic; 2 International Institute of Tropical Agriculture, Kampala, Uganda; 3 Institute of Experimental Botany, Centre of the Region Haná for Biotechnological and Agricultural Research, Olomouc, Czech Republic; 4 Laboratory of Tropical Crop Improvement, Division of Crop Biotechnics, Katholieke Universiteit Leuven, Leuven, Belgium; 5 Bioversity International, Leuven, Belgium; 6 International Institute of Tropical Agriculture, Arusha, Tanzania; Universidad de Colima Facultad de Ciencias Biologicas y Agropecuarias, MEXICO

## Abstract

Banana (*Musa spp*.) is an important crop in the African Great Lakes region in terms of income and food security, with the highest per capita consumption worldwide. Pests, diseases and climate change hamper sustainable production of bananas. New breeding tools with increased crossbreeding efficiency are being investigated to breed for resistant, high yielding hybrids of East African Highland banana (EAHB). These include genomic selection (GS), which will benefit breeding through increased genetic gain per unit time. Understanding trait variation and the correlation among economically important traits is an essential first step in the development and selection of suitable GS models for banana. In this study, we tested the hypothesis that trait variations in bananas are not affected by cross combination, cycle, field management and their interaction with genotype. A training population created using EAHB breeding material and its progeny was phenotyped in two contrasting conditions. A high level of correlation among vegetative and yield related traits was observed. Therefore, genomic selection models could be developed for traits that are easily measured. It is likely that the predictive ability of traits that are difficult to phenotype will be similar to less difficult traits they are highly correlated with. Genotype response to cycle and field management practices varied greatly with respect to traits. Yield related traits accounted for 31–35% of principal component variation under low and high input field management conditions. Resistance to Black Sigatoka was stable across cycles but varied under different field management depending on the genotype. The best cross combination was 1201K-1xSH3217 based on selection response (R) of hybrids. Genotyping using simple sequence repeat (SSR) markers revealed that the training population was genetically diverse, reflecting a complex pedigree background, which was mostly influenced by the male parents.

## Introduction

East Africa is considered a secondary center of banana genetic diversity. Uganda in particular is a home to over eighty cultivars of East African Highland banana (EAHB) commonly divided into cooking and beer types [1]. The crop greatly contributes to the income and food security of many smallholder farmers in the region. The significance of the crop in the region is reflected in the per capita consumption that ranges between 250kg and 600kg with an average of 400kg in Uganda [2]. Over 85% of the production is consumed locally due to high demand [3, 4]. Sustainable production of bananas is a challenge because of disease, insect and nematode pressure. This is worsened by abiotic stress arising through factors associated with climate change [5]. Yield reductions in EAHB are caused by pests such as root burrowing nematodes especially *Radopholus similis* and banana weevil (*Cosmopolites sordidus*). Black Leaf Streak (Black Sigatoka), a fungal disease caused by *Mycosphaerella fijiensis* reduces the photosynthetic area of the plant, which decreases yield. Banana bacterial wilt caused by *Xanthomonas campestris* pv. *musacearum* causes 100% yield loss when the banana is attacked [6–8]. Variation in rainfall patterns impacts banana production by causing drought stress because most farmers in the region rely on rain for agricultural production. Although phenotypic variation is observed in EAHB, their genetic variation is low [9, 10] making them all susceptible to biotic and abiotic stress. Adaptation of cultivated banana varieties to changing environment is limited because while some are capable of sexual reproduction, they are all propagated clonally.

**Fig 1:**
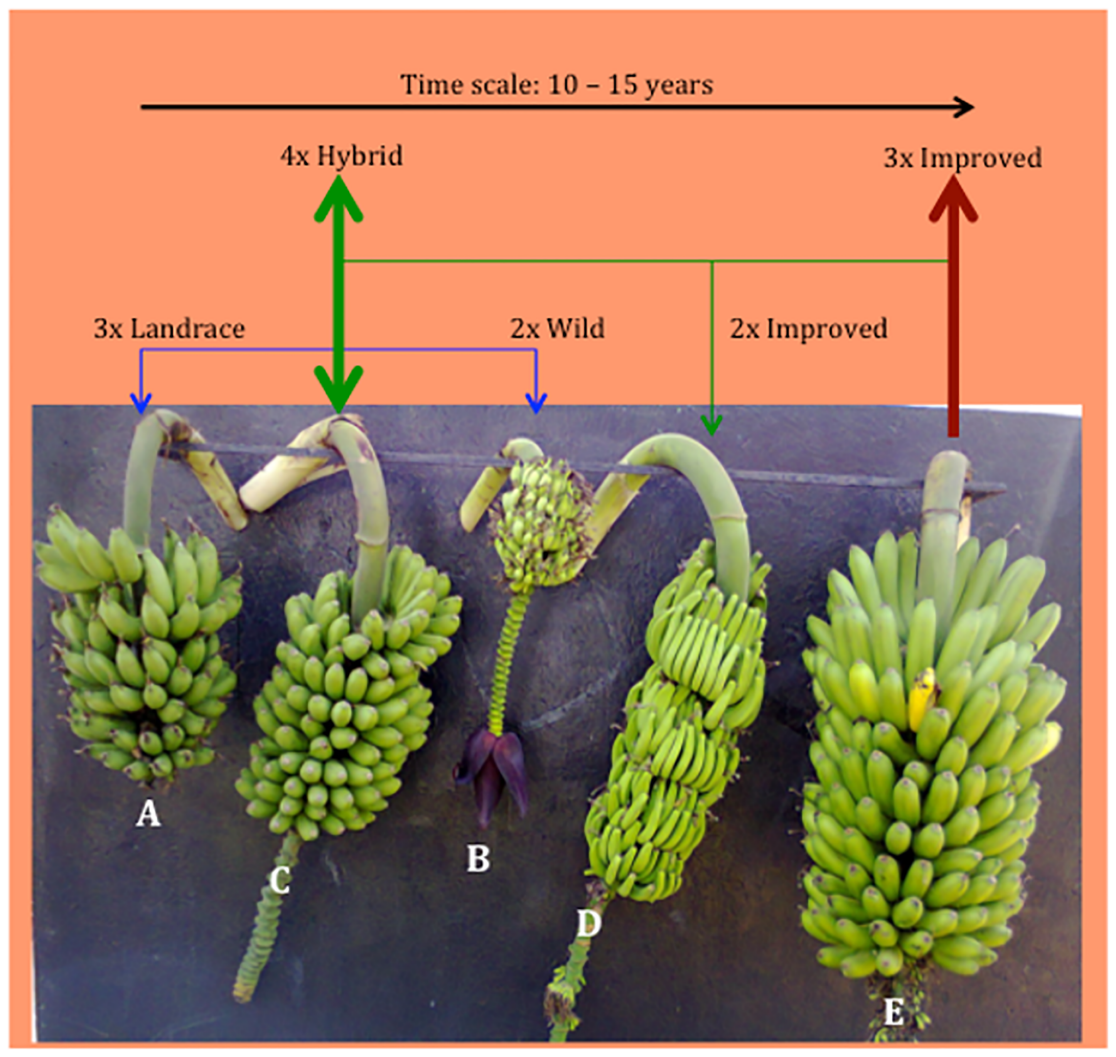
Conventional banana breeding starts with crossing 3x inferior and parthenocarpic landrace varieties (A) with a wild, seeded 2x accession (B). 4x resulting from this cross (C) are selected and crossed with improved 2x hybrids (D). The resulting secondary 3x (E) are selected and evaluated as potential improved varieties. This process takes up to 15 years.

In order to meet the food demand for the growing population, breeding for resistance and high yielding varieties is considered to be the most sustainable solution to address banana production constraints [11, 12]. Unlike other crops, banana breeding is complicated by the polyploid nature of the crop characterized by abnormal meiosis in the cultivated triploid varieties that results in reduced fertility or complete sterility [13–15]. Crossing cultivated varieties with resistant wild diploids is possible, but a majority of the generated hybrids are inferior due to linkage drag of unfavorable genes from the wild diploids. However, when tetraploids are obtained, further improvement is possible because they are both male and female fertile (Fig 1). Incorporating resistance while maintaining the unique attributes such as fruit colour, aroma, texture and taste in existing varieties is a big challenge to banana breeders that calls for dedicated effort and careful choice of cross combinations. Crossbreeding is labour-intensive, costly and time consuming. In the last two decades, some success has been registered with new hybrids released to farmers while others are in the advanced stages of evaluation [16]. In order to keep up with the pace at which environmental changes occur and the demand for new varieties that are productive and of good quality, new breeding strategies should be employed to increase breeding efficiency and reduce the lengthy selection period [3].

Marker assisted selection (MAS) has been implemented in many animal and crop breeding programs. The success of MAS greatly depends on the genetic architecture of traits being improved. To date MAS has not been effectively deployed in banana breeding. The possible reasons are polyploidy, important economic and agronomic traits may be controlled by many quantitative trait loci (QTL), each with a small additive effect, and the lack of saturated linkage maps for QTL mapping. It is believed that the application of genomic selection (GS) will improve the efficiency of crossbreeding programs especially for crops with long breeding and selection cycle [17, 18] like banana. GS is a form of MAS where selection is based on the genomic estimated breeding values (GEBV) of superior individuals in the population as determined by a statistical model [19–21]. This technique is well established in animal breeding [22, 23]. In plants, GS has been tested in maize and wheat [24], white spruce [25], rice [26] and cassava [27]. However, in bananas GS is in its infancy. Given that new varieties are selected based on a combination of traits, a selection index of GEBV in bananas is necessary.

GS studies have reported varying accuracies in prediction (predictive ability of GS models) and this has been attributed to differences in trait heritability, number of markers, training population size and genotype x environment interaction [24]. Bananas as perennial plants suffer the consequences of nutrient deficiency and soil moisture variation across seasons and locations depending on field management practices. Breeding generates genotypes from many crosses that are genetically different and respond to growth environment differently and this could affect the accuracy of GS. Therefore, understanding trait variation and the correlation between different traits is essential to guide the development and selection of suitable GS models for banana breeding. In this study we tested the hypothesis that trait variations in bananas are not affected by cross combination, cycle, field management and their interaction with genotype. For this, a training population created using EAHB breeding material and its progeny was phenotyped in two contrasting conditions. Genetic diversity of the training population was assessed using simple sequence repeat (SSR) markers.

## Materials and methods

### Plant population

Data were collected at the International Institute of Tropical Agriculture, Uganda from a banana genomic selection (GS) training population between 2013 and 2016. The institute is located at Namulonge research station, 0.53° N 32.58° E, 1150 m above sea level with rainfall of about 1200 mm/y split into two rainy seasons, March-June and September-December and an average annual temperature of 22°C. The GS population consisted of 307 genotypes that included diploid (11%), triploid (85%) and tetraploid (4%) plants (S1 Table). The ploidy level of the genotypes was determined using flow cytometry [28, 29]. The core breeding lines (parents) accounted for 12% of the entire population. Two fields were established with each genotype replicated three times in a completely randomized design. Suckers were used as planting materials and before planting, 20kg of farmyard manure was applied in each hole. One field (GS1) was managed without mulching, additional manure nor inorganic fertilizer (low input). The second field (GS2) was mulched twice a year. Six months after planting, 480 g of NPK (25:5:5) fertilizer was added and the same amount was added to each mat per year (high input).

### Traits

The yield-related traits scored included: days to fruit maturity (DFM) that is, days between flowering and harvesting, bunch weight at full maturity (BWT), number of hands (cluster) (NH) and number of fruit fingers (NF), fruit length (FL), fruit circumference (FC), fruit diameter (FRD), pulp diameter (PLD) and peel thickness (PED), where PED = (FRD—PLD)/2. The vegetative (growth) traits included: number of standing leaves at flowering (NSLF), youngest leaf spotted with Black Sigatoka at flowering (YLSF), index of non-spotted leaves at flowering (INSL), height of tallest sucker at harvesting (HTSH), plant height at flowering (PHF), plant girth at 100 cm from soil surface (PG), height of tallest sucker at flowering (HTSF), total number of suckers at flowering (TS), number of standing leaves at harvesting (NSLH) and youngest leaf spotted with black sigatoka at harvesting (YLSH).

Total number of suckers (TS) was recorded at flowering in cycle 1 only after which each mat was left with a maximum of three plants and these included the flowered plant, follower sucker and the sucker produced by follower sucker if present. A Vernier caliper was used to measure FRD and PLD. Fruit related traits such as FL, FC, FRD and PLD were recorded from the middle finger of the second hand on the bunch. Measurements for FC, FRD and PLD were recorded midway the length of the finger. However, to measure FRD and PLD, a cross-section of the fruit was made to expose the pulp. The INSL was calculated from the formula, INSL = 100*(YLSF-1)/NSLF [30]. This formula should give percentage values ranging from 0–100% to represent completely susceptible (0%) and completely resistant (100%). In order to get 100% INSL for completely resistant genotypes, the YLSF was scored as NSLF +1 thus INSL = 100*((NSLF+1)-1)/NSLF or INSL = 100*NSLF/NSLF

### Data analysis

All analyses were performed in R, open source statistical software from www.r-project.org. A combination of Shapiro-Wilk test, boxplots, standard deviations and histograms were used to check for normality and outliers in the data and where necessary the outliers were removed before further analysis. Total number of suckers and bunch weight were transformed by square root. Using the aggregate function from library (plyr), trait means were calculated for every genotype and cross combination (family) in every cycle, and field and these were used in correlation analysis and principal component analysis (PCA).

Correlation analysis and test of significance for the correlations between traits were done using library (Hmisc) and Student’s t-test based on cycle 2 data for cross combinations. Coefficient of determination (R^2^) was calculated as a square of correlation coefficient between cycle 1 and 2 data. To understand the structure of the population and how different traits influenced that structure, principal component analysis was done using PCA function provided in the library (FactoMineR). Traits (dependent variable), cross combinations and individual genotypes were projected on the first two components (Dim1 and Dim2).

Sources of trait variation were assessed using unbalanced analysis of variance (ANOVA) based on cycle 1 and 2 data. Linear models were constructed for each trait in respect to each cycle, field management practice and their interaction with genotype as model_fit = lm(trait response~clone+cycle+field+clone:field+clone:cycle, data = mydata) where lm = linear model function. Type III SS ANOVA tables were generated using Anova function provided in the library(car) as result = Anova(model_fit, singular.ok = TRUE, type = “III”). In cases where no significant interactions were observed between two independent variables and where one explanatory variable was not significant, then type II or type I SS ANOVA was used for further investigation.

Selection differential (S) and response to selection (R) were used to compare performance of parental cross combinations [31]. S and R were calculated as, S = P—G and R = H—G, where P = average performance of a pair of parents, G is the average performance of all parental lines in the training population and H is the average performance of all hybrid that shared same parental pair. Only cross combinations that had at least five hybrids were compared across all traits using combined data from the two fields.

### Genetic diversity

Genetic diversity of the training population was assessed using simple sequence repeat (SSR) markers. Cigar leaf samples were collected from the training population in Uganda and shipped to the Institute of Experimental Botany, Olomouc, Czech Republic under cold chain. Samples were lyophilized prior to DNA extraction. DNA from lyophilized samples was extracted using NucleoSpin Plant II kit, Macherey-Nagel, Germany, following the manufacturer’s instructions. The concentration and quality of DNA was assessed by NanoDrop ND-1000 spectrophotometer. Nineteen informative *Musa* SSR primers were used to genotype the GS training population. The list of primers used, polymerase chain reaction (PCR) conditions, and fragment analysis procedure were adopted from Christelová et al. [32].

Two independent rounds of PCR were performed on each sample. The concordance of alleles from each sample were inspected and scored manually in GeneMarker v1.75 (Softgenetics, State College, PA, USA). A third round of PCR was performed only for samples that showed incongruity with the two reactions. Alleles were scored as dominant markers for presence and absence (1/0). Data were imported in R and squared Euclidean distances were generated using the function dist provided in the library(ape). Clustering was done with function hclust based on ward.D method [33, 34]. Polymorphism information content of each marker was estimated by PowerMarker v3.25 software [35].

## Results

During data analysis, some genotypes were excluded for some traits due to missing data or extreme outliers. The outliers were mainly recorded on plants that were infected with banana *Xanthomonas* wilt before full maturity, plants that snapped due to weevil damage and premature breaking of the peduncle due to windstorm.

### Correlation of traits

Significant correlations were observed among and between growth and yield traits (Tables 1 and 2). PHF had significant positive correlation with PG followed by HTSF. PG positively correlated with BWT, NF and HTSF in that respective order. The traits associated with Black Sigatoka resistance (NSLF, YLSF and INSL) also correlated significantly to each other. However, they had significant negative correlations with fruit traits such as FC, FRD and PLD. A positive and significant correlation was observed between BWT and all fruit traits (NH, NF, FL, FC, FRD, PLD), which were similarly significantly and positively correlated to each other. Under conditions of low input field management (GS1), TS, NSLH and NF were not significantly correlated with other traits while under high input field management (GS2), it was INSL, DFM and HTSH that did not have significant correlation with other traits. In both fields, the highest positive correlations were observed among the yield traits. In this population, absolute apical dominance was not observed as all genotypes had at least one sucker at the time of flowering. However, sucker regulation varied among genotypes with a range of 1–25 suckers per plant.

**Table 1:** Pearson’s correlation coefficients of traits under low input field management (GS1).

	**PHF**	**PG**	**NSLF**	**YLSF**	**HTSF**	**TS**	**INSL**	**DFM**	**NSLH**	**BWT**	**NH**	**NF**	**FL**	**FC**	**FRD**	**PLD**	**PED**
**PHF**																	
**PG**	0.807***																
**NSLF**	0.108	0.254															
**YLSF**	0.083	0.181	0.926***														
**HTSF**	0.373**	0.36**	0.319**	0.364**													
**TS**	−0.229	−0.329**	0.022	0.116	0.3**												
**INSL**	−0.001	0.021	0.579***	0.834***	0.326**	0.24											
**DFM**	0.086	0.133	0.282	0.277**	0.109	0.038	0.231										
**NSLH**	0.042	0.078	0.386	0.352**	0.363**	0.034	0.194	-0.356									
**BWT**	0.346**	0.554***	−0.083	−0.122	0.213	0.02	−0.133	0.152	−0.22								
**NH**	0.372**	0.426**	0.19	0.166	0.087	0.041	0.119	0.191	−0.021	0.411**							
**NF**	0.412**	0.512***	0.226	0.195	0.151	−0.032	0.126	0.221	0.053	0.4**	0.878***						
**FL**	0.2	0.411**	−0.077	−0.113	0.057	−0.042	−0.123	0.173	−0.266	0.855***	0.168	0.169					
**FC**	0.191	0.375**	−0.284**	−0.338**	0.025	−0.097	−0.323**	0.005	−0.254	0.807***	0.019	0.008	0.856***				
**FRD**	0.206	0.359**	−0.357**	−0.415**	0.022	−0.11	−0.395**	−0.055	−0.258	0.782***	0.017	0.02	0.82***	0.987***			
**PLD**	0.192	0.333**	−0.379**	−0.432**	−0.004	−0.133	−0.41**	−0.099	−0.199	0.717***	0.048	0.057	0.709***	0.9***	0.919***		
**PED**	−0.26**	0.108	0.143	0.024	0.007	−0.078	−0.114	0.108	−0.013	0.225	−0.182	−0.118	0.359**	0.293**	0.272**	0.179	

**Table 2:** Pearson’s correlation coefficients of traits under high input field management (GS2).

	**PHF**	**PG**	**NSLF**	**YLSF**	**HTSF**	**TS**	**INSL**	**DFM**	**NSLH**	**BWT**	**NH**	**NF**	**FL**	**FC**	**FRD**	**PLD**	**PED**
**PHF**																	
**PG**	0.774***																
**NSLF**	−0.422**	−0.257															
**YLSF**	−0.197	−0.024	0.75***														
**HTSF**	0.702***	0.563***	−0.357**	−0.251													
**TS**	0.358**	0.224	−0.092	0.062	0.45***												
**INSL**	0.213	0.272	−0.128	0.548***	0.084	0.197											
**DFM**	−0.007	0.006	−0.026	0.063	−0.218	−0.02	0.152										
**NSLH**	−0.149	−0.077	0.619***	0.533***	−0.222	−0.156	0.002	−0.194									
**BWT**	0.37**	0.623***	−0.081	−0.14	0.46***	0.165	−0.132	0.019	−0.173								
**NH**	0.218	0.424**	0.071	0.119	0.227	0.09	0.095	0.175	−0.068	0.521***							
**NF**	0.368**	0.582***	0.006	0.11	0.348**	0.169	0.194	0.227	−0.007	0.57***	0.843***						
**FL**	0.204	0.439**	−0.076	−0.145	0.285**	0.134	−0.151	−0.065	−0.22	0.826***	0.284**	0.27**					
**FC**	0.327**	0.449**	−0.233	−0.255	0.397**	0.198	−0.146	−0.151	−0.19	0.807***	0.148	0.153	0.85***				
**FRD**	0.39**	0.478**	−0.254	−0.281**	0.42**	0.28**	−0.156	−0.154	−0.223	0.791***	0.158	0.184	0.803***	0.968***			
**PLD**	0.389**	0.446**	−0.271	−0.3**	0.398**	0.31**	−0.161	−0.176	−0.22	0.741***	0.114	0.135	0.76***	0.945***	0.991***		
**PED**	0.005	0.199	0.062	0.022	0.242	−0.171	−0.048	0.022	−0.077	0.513***	0.324**	0.34**	0.464**	0.337**	0.217**	0.1	

*** P-value < 0.001,

** P-value < 0.05 but > 0.001

### Principal component analysis (PCA)

**Fig 2:**
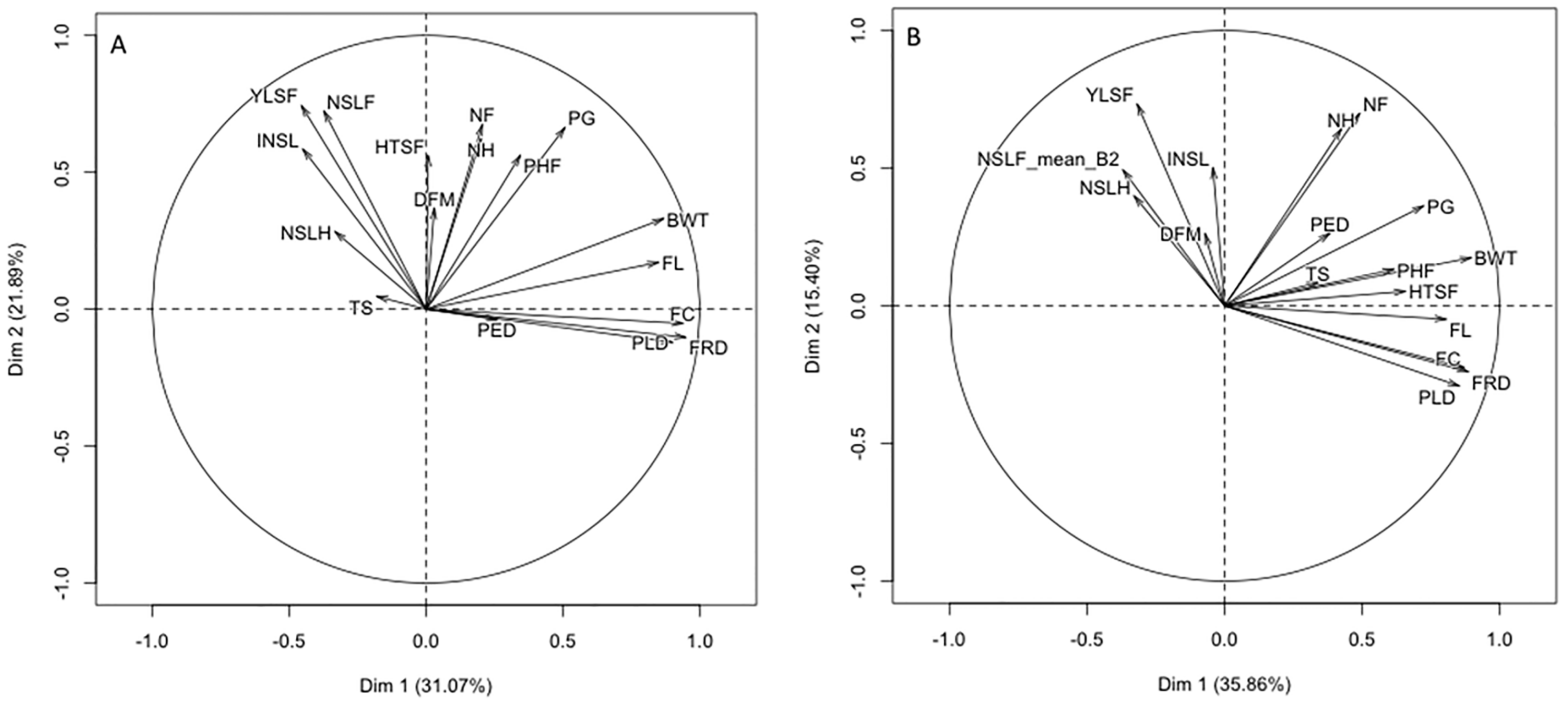
Principal component analysis plots generated in R using package FactoMineR for the traits scored in a banana genomic selection training population. (A) shows the distribution of traits under low input field management (GS1) and (B) shows the distribution of traits under high input field management (GS2) on the first two components.

**Fig 3:**
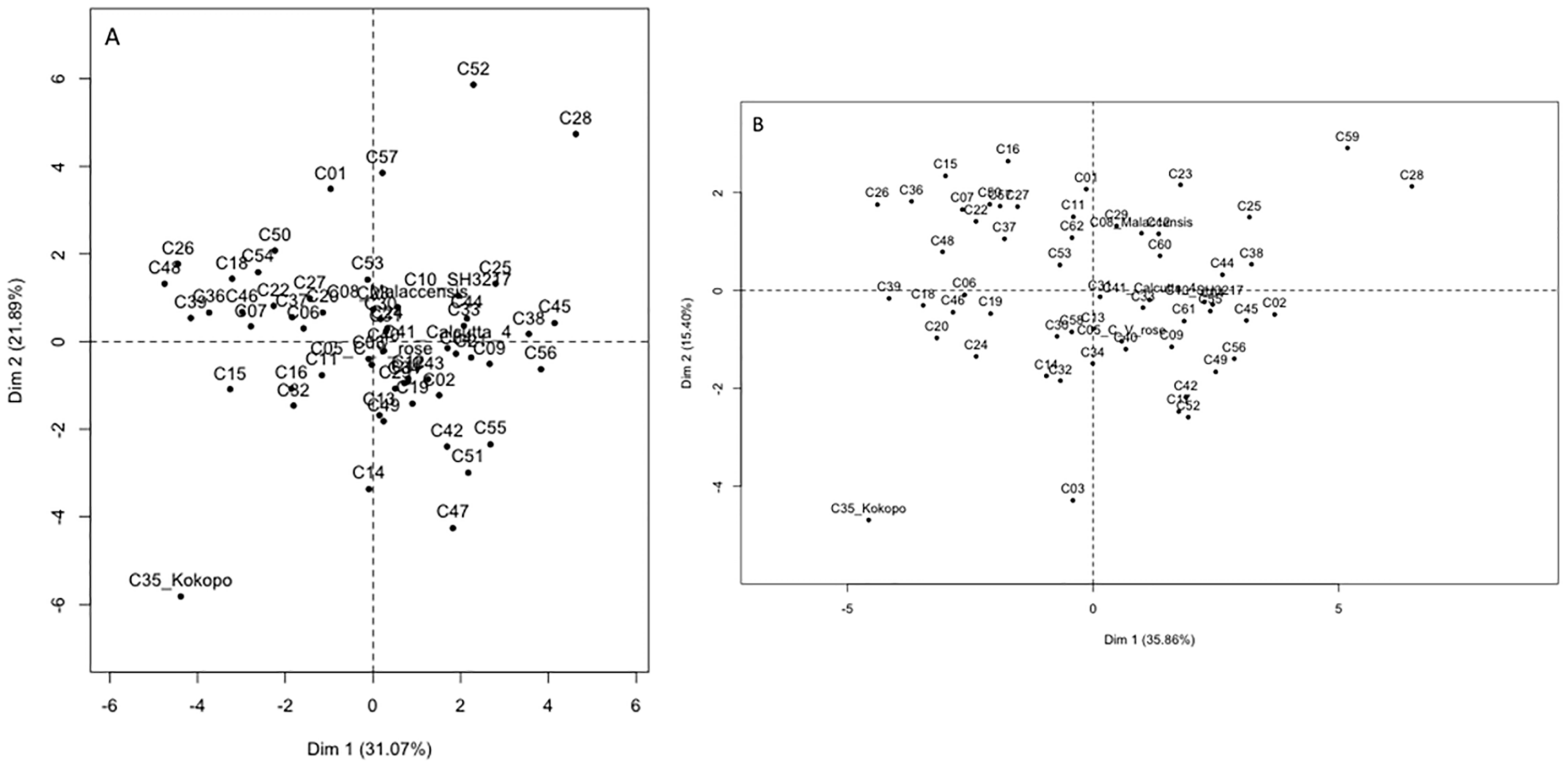
Principal component analysis plots generated in R using package FactoMineR for the cross combinations in a banana genomic selection training population. (A) shows the distribution of cross combinations under low input field management (GS1) and (B) shows the distribution of cross combinations under high input field management (GS2) on the first two components.

Principal component analysis showed that in both fields, the yield (fruit) traits contributed to the first component (Dim 1) while the vegetative (growth) traits contributed to the second component (Dim 2) (Fig 2A and 2B). Among the vegetative traits, PHF and PG contributed to Dim 1. Dim 1 accounted for 31.07% of variation in GS1 and 35.86% in GS2. Dim 2 accounted for 21.89% of variation in GS1 and 15.40% in GS2. The traits with the highest negative loading on Dim 1 included FC, FRD and PLD for GS1 while for GS2 it was FC, FRD, PLD and FL. In both GS1 and GS2, the traits with the highest positive loading on Dim 2 were NSLF, YLSF, INSL and NSLH. Both DFM and TS had the least contribution to the two components with completely different orientation in GS1 and GS2. Generally, in both fields the two components accounted for 50% of the variation observed in the genotype cross combinations (Fig 3A and 3B).

**Fig 4:**
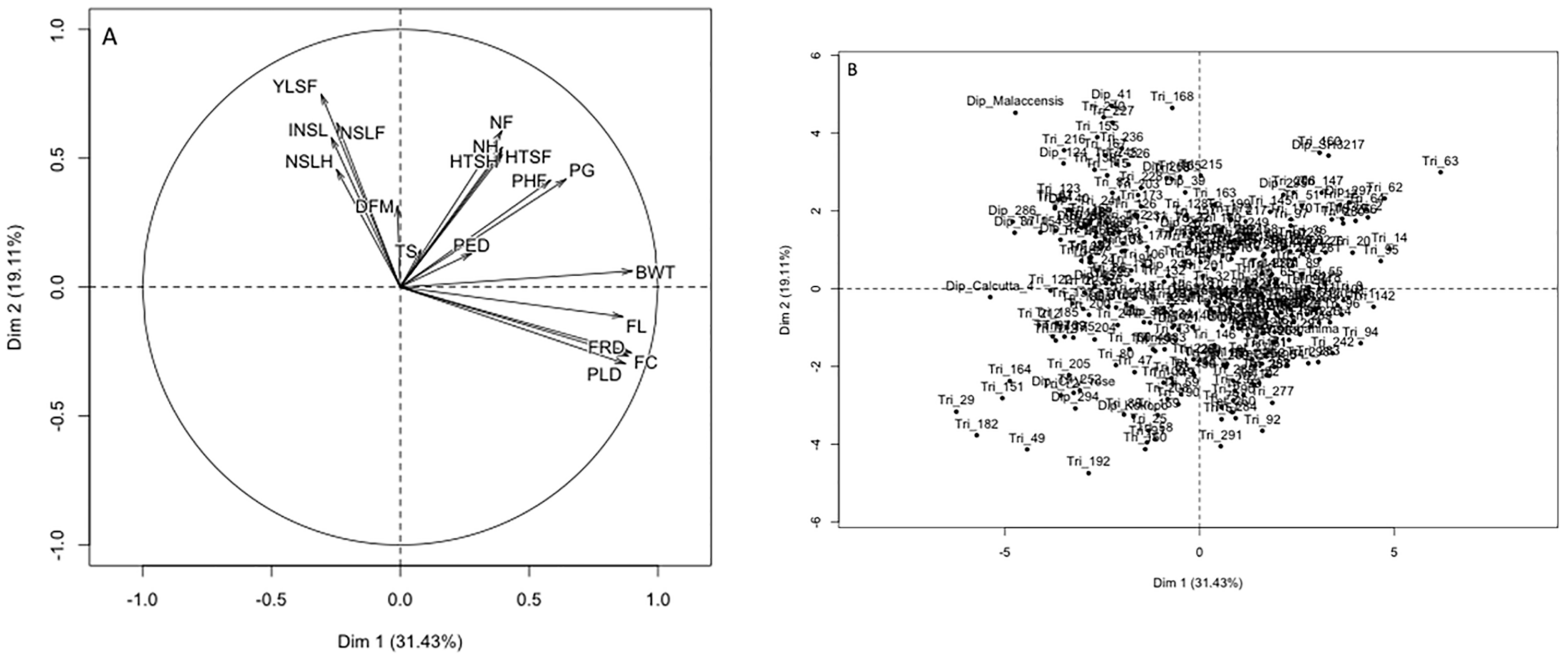
Principal component analysis plots generated in R using package FactoMineR for the traits scored in a banana genomic selection training population. (A) shows the distribution of traits for individual genotypes and (B) shows the distribution of individual genotypes on the first two components based on mean of combined data from the two fields.

For individual genotypes, a similar trend was observed with Dim 1 and Dim 2 accounting for 31.43% and 19.11% of total trait variation, respectively (Fig 4A). Projection of the individual factors (genotypes) on the two components did not reveal any distinct population structure (Fig 4B). The same trend was observed when individual cross combinations were projected on the two components. However, in GS1 cross combinations C35 (917K-2 x Kokopo), C28 (8817S-1 x 917K-2) and C52 (SH2095 x SH2766) and in GS2 cross combinations C35 (917K-2 x Kokopo), C22 (365K-1 x 660K-1) and C29 (8817S-1 x 917k-2) were distinct and clearly separated out from the others (Fig 3a and 3b). When the data were re-examined, genotypes from cross C35 had the least average scores on the yield traits while cross C22, C29 and C52 had the highest average scores on the yield traits. All the four planes of the two components were represented in the population.

Based on Black Sigatoka resistance and fruit filling (indicated by FRD), four main groups were represented in the population: (i) genotypes with high INSL and good fruit filling, (ii) high INSL with poor fruit filling, (iii) low INSL with good fruit filling and (iv) low INSL with poor fruit filling. On average the observed INSL and FRD for the genotypes in the four groups were as follows: (i) 78.1% and 3.0cm, (ii) 80.1% and 1.4cm, (iii) 66.8% and 3.1cm, and (iv) 67.1% and 1.4cm, respectively. Genotypes projected on Dim 2 had high average scores on NSLF, YLSH, INSL, and NSLH and in contrast they had the lowest average scores on BWT, FL, FC, FRD, and PLD and the reverse was true for those projected on Dim 1.

### Analysis of variance

**Fig 5:**
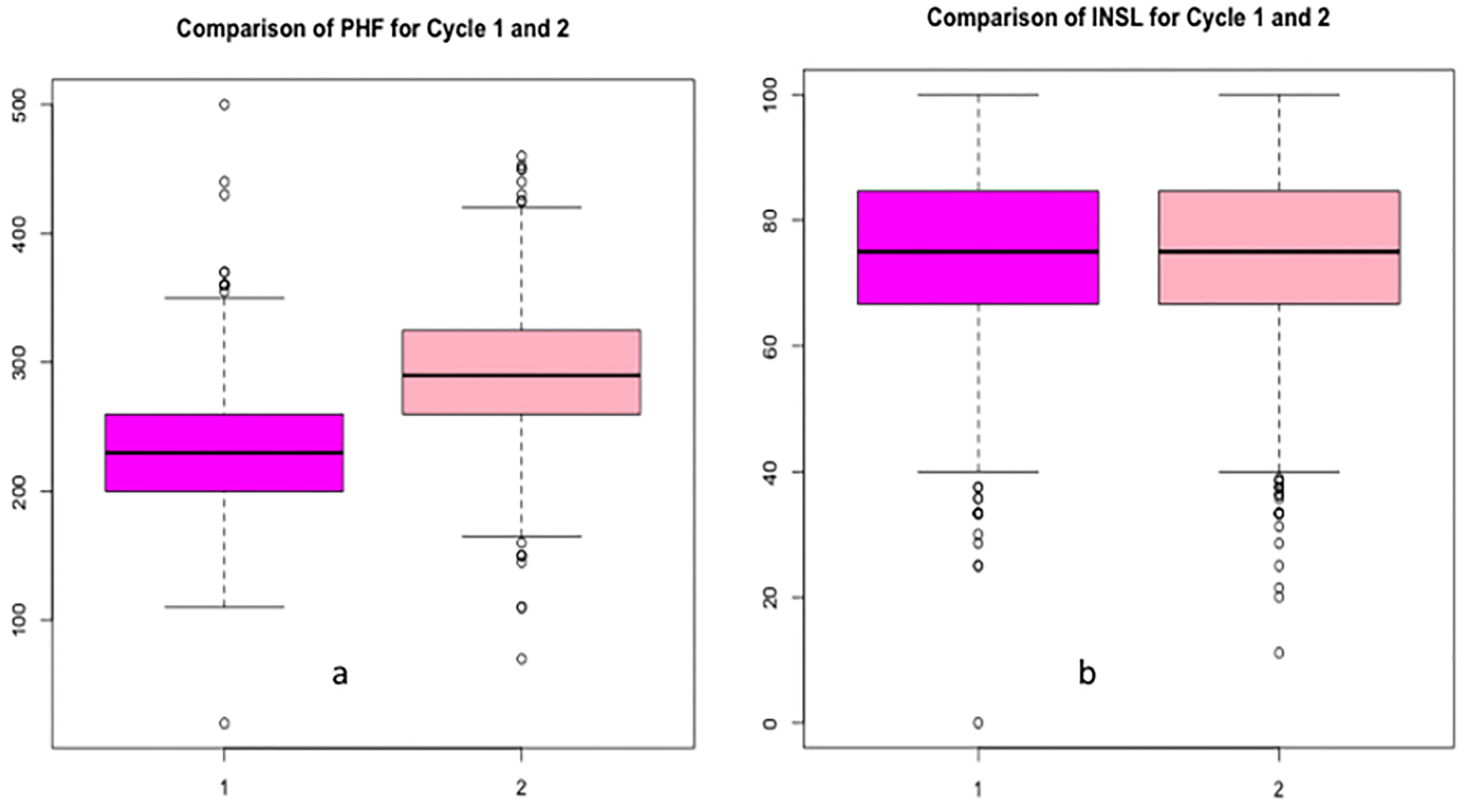
Effect of cycle on trait variation in bananas, where (a) shows an increase in plant height at flowering at cycle 2 while (b) shows no increase in index of non-spotted leaves at cycle 2.

Visual inspection of boxplots for various traits indicated a cycle effect on data distribution of some traits while others were not affected by cycle. For example, Plant height increased at cycle 2 while index of non-spotted leaves did not increase (Fig 5a and 5b) and this was confirmed by ANOVA results. Fruit traits such as FC, FRD and PLD showed a bimodal distribution with the histogram having two peaks. Based on these parameters, the population could be separated into two main groups, poor fruit filling genotypes with FRD < 2.0 cm and FC < 8.0 cm, and good fruit filling genotypes with FRD ≥ 2.0 cm and FC ≥ 8.0 cm (S1A–S1D Fig).

Coefficients of determination showed that under low input, cycle had less effect on NSLF, YLSF, INSL, TS, HTSF and PED across genotype cross combinations (Table 3). The Student’s t-test revealed that both PED and HTSF were the most stable traits across cycles at 95% confidence level with P = 0.515 and P = 0.108, respectively. Under high input, cycle accounted for less than 50% of the variation in NSLF, YLSF, INSL, TS, HTSF, DFM, NSLH, NH, NF and PED between cross combinations. Just as in the first field, PED and HTSF were the least affected with P = 0.216 and P = 0.108, respectively. Under high input field management, trait variation due to cycle was more homogenous as compared to low input field management. However, in both cases the effects were statistically significant (P < 0.001) indicating that cycle is a source of variation in genotype performance.

**Table 3:** Coefficient of determination and Student’s t-test P-values showing the effect of cycle on cross combinations.

	GS1			GS2		
Traits	df	R^2^	P-value	df	R^2^	P-value
NH	60	0.87	<0.0001	56	**0.44**	<0.0001
PLD	57	0.78	<0.0001	56	0.65	<0.0001
FRD	59	0.77	<0.0001	56	0.68	<0.0001
PED	58	**0.06**	**0.5150**	56	**0.03**	**0.2161**
BWT	60	0.79	<0.0001	56	0.74	<0.0001
NF	60	0.54	<0.0001	56	**0.37**	<0.0001
FL	59	0.77	<0.0001	56	0.64	<0.0001
FC	58	0.79	<0.0001	56	0.73	<0.0001
DFM	59	0.54	<0.0001	56	**0.25**	<0.0001
NSLH	60	0.63	<0.0001	56	**0.38**	<0.0001
PHF	66	0.65	<0.0001	63	0.73	<0.0001
PG	66	0.65	<0.0001	63	0.73	<0.0001
NSLF	66	**0.25**	<0.0001	63	**0.28**	<0.0001
YLSF	66	**0.47**	<0.0001	63	**0.26**	<0.0001
INSL	66	**0.14**	0.0015	63	**0.21**	0.0001
TS	68	**0.12**	0.0032	68	**0.12**	0.0032
HTSF	68	**0.04**	**0.1084**	68	**0.04**	**0.1084**

Df = degrees of freedom, GS1 = low input field, GS2 = high input field and R^2^ = coefficient of determination

When generating ANOVA models, genotype (clone) was assumed to be the main source of variation. In addition to genotype the effect of cycle, field and their interaction with genotype was investigated. In all models for all traits, genotype had significant effect on trait variation with P < 0.001 (Table 4, S3 Table). Traits that were not affected by the interaction between genotype and field management practice include PHF and PG whereas traits not affected by interaction between genotype and cycle include NSLF, YLSF, INSL, YLSH, FL, FRD and PED (P > 0.05). Weak interaction between genotype and cycle was observed on NSLH and HTSH with P = 0.0417 and 0.0408, respectively. In some cases, although there were significant interactions between genotype and field or cycle, either field or cycle did not show significant effect on the trait when interaction was included in the model.

**Table 4:** Effect of genotype (clone), field management, cycle and their interaction on trait variation.

Dep. variable	Indep. variable	Sum Sq	Df	F value	Pr(>F)
PHF	Clone	2222889.11	306	3.77	<0.0001
	Clone:Field	432297.46	284	0.79	0.9947
	Clone:Cycle	332846.71	299	1.05	0.2662
PG	Clone	73176.82	306	4.30	<0.0001
	Clone:Field	12061.30	284	0.76	0.9981
	Clone:Cycle	13057.24	299	1.51	<0.0001
INSL	Clone	116602.02	306	2.44	<0.0001
	Clone:Field	58583.77	284	1.32	0.0005
	Clone:Cycle	51026.49	299	0.95	0.6947
TS^sqrt^	Clone	240.28	305	3.21	<0.0001
	Clone:Field	100.88	282	1.46	<0.0001
BWT^sqrt^	Clone	1213.89	303	12.55	<0.0001
	Clone:Field	126.77	269	1.48	<0.0001
	Clone:Cycle	108.68	276	1.49	<0.0001
FC	Clone	9506.06	300	16.11	0.0000
	Clone:Field	733.66	269	1.39	0.0001
	Clone:Cycle	751.00	272	1.29	0.0021
PLD	Clone	865.42	299	17.60	0.0000
	Clone:Field	68.27	269	1.54	<0.0001
	Clone:Cycle	60.55	271	1.29	0.0022
PED	Clone	20.96	299	11.41	<0.0001
	Clone:Field	16.61	269	10.05	<0.0001
	Clone:Cycle	3.15	271	0.80	0.9913

^sqrt^ Original data transformed by square root

Whereas there were significant interactions between genotype and field management, there was no significant main effect of field on NSLF, YLSF, HTSF, INSL, TS, NSLH, YLSH, HTSH, NH, NF and PED. Similarly, in the presence of significant interaction between genotype and cycle, there was no main effect of cycle on INSL, HTSF, HTSH, FC, PLD and PED (Table 4, S3 Table). When the interactions were removed from the models, all the factors had significant effect on the traits except INSL and PED, for which cycle had no effect. Analysis was repeated on these two traits using type I and type II ANOVA and both produced similar results as that observed with type III SS.

### Performance of cross combinations (parental pairs)

The GS training population consisted of 77 different cross combinations representing about two decades of banana breeding activities by IITA and NARO Uganda. Some of these cross combinations gave rise to the tetraploids and improved diploids that are part of the core breeding lines in the program. Tetraploids and triploids were predominantly used as female parents while the diploids provided the pollen source but in some instances 2x by 2x or 4x by 4x crosses were made. The majority of the cross combinations were excluded for this analysis in this work because they had less than five hybrids in the population. However, crosses between different EAHB with Calcutta 4 were treated as one cross because the EAHB represent a clone set with very low genetic diversity [9]. In total sixteen cross combinations were compared and they included one 2x by 2x, one 3x by 2x and fourteen 4x by 2x crosses (Table 5 and S2 Table).

**Table 5:** Comparison of mean performance of parental cross combinations (S) and hybrids from those crosses (R) against the mean of all parents.

CROSS	C04	C05	C08	C10	C11	C12	C13	C16	C22	C27	C31	C33	C34	C37	C61	MxC4
S (NSLF)	-0.5	-0.2	1.2	0.4	0.2	-0.2	-0.3	1.2	0.7	1.9	0.1	-0.3	-0.1	1.4	0.4	-1.1
R (NSLF)	-0.4	0.5	0.8	0.0	1.4	0.9	0.1	**1.8**	2.1	**1.8**	0.6	0.3	-0.2	1.4	0.8	0.1
S (YLSF)	-0.7	-0.4	1.4	0.3	0.0	-0.2	-0.3	1.7	0.2	1.7	-0.8	-1.1	-0.7	1.1	-0.3	-1.5
R (YLSF)	-0.7	0.3	0.7	0.0	0.8	0.4	0.1	1.8	**2.2**	1.7	0.4	-0.1	-0.1	1.2	0.7	-0.1
S (PHF)	24.1	-33.5	6.6	35.2	35.8	17.2	-37.5	3.8	-21.8	-1.5	-14.0	-11.4	-58.2	-22.5	0.2	25.9
R (PHF)	14.8	-23.8	10.1	**33.6**	-23.4	-7.4	-39.4	-6.6	-62.3	2.5	0.5	7.9	-31.0	-17.6	-9.5	7.6
S (PG)	9.6	-2.9	5.0	11.1	**11.7**	2.8	-7.5	-0.1	-5.3	1.3	0.9	1.4	-8.5	-1.6	3.6	3.3
R (PG)	3.6	-3.2	1.2	6.0	-1.4	2.3	-6.8	-1.4	-5.7	-0.6	2.2	4.9	-5.4	-2.0	3.3	2.0
S (HTSF)	11.4	-8.5	30.1	24.2	31.7	-5.5	-18.1	20.5	-46.3	23.0	-27.5	-25.0	-33.7	0.3	-4.7	23.0
R (HTSF)	15.0	-10.3	6.3	**23.3**	-21.1	-7.3	-26.8	14.3	-32.5	13.4	0.8	-2.5	-14.4	-4.0	-6.4	4.2
S (INSL)	-1.8	-1.0	4.9	0.9	0.3	0.2	0.7	7.2	-1.5	3.9	-6.4	-6.5	-4.2	1.1	-4.3	-7.0
R (INSL)	-2.9	0.6	1.4	1.2	-1.9	-0.7	1.1	5.7	**7.2**	4.1	0.1	-1.8	1.8	2.7	2.2	-0.9
S (TS)	-1.6	2.8	0.7	-1.0	1.1	-1.1	3.0	1.2	-1.7	0.1	-3.3	-2.9	1.3	-0.7	-0.4	0.0
R (TS)	-0.3	**1.9**	0.6	0.8	-1.0	-1.2	0.8	1.0	-0.4	-0.8	0.3	-1.9	1.2	0.0	0.7	-1.2
S (DFM)	2.4	2.7	15.9	10.0	-1.3	4.9	6.5	31.4	14.2	32.9	8.9	10.9	8.8	28.0	8.2	-21.3
R (DFM)	7.8	6.3	21.1	7.3	-1.9	19.9	1.6	8.3	**54.6**	32.6	23.9	11.2	13.5	22.3	20.7	7.2
S (NSLH)	-0.7	-0.9	0.3	-0.4	-0.5	-0.1	-0.7	1.3	0.5	1.5	0.4	0.1	-0.3	1.4	0.6	-0.7
R (NSLH)	-0.9	0.0	0.8	-0.4	1.5	0.6	0.1	**2.3**	0.3	1.4	0.3	0.1	-0.1	1.1	0.1	0.2
S (YLSH)	-0.4	-0.4	0.3	-0.1	-0.2	0.0	-0.3	1.0	0.1	1.1	-0.1	-0.2	-0.3	0.6	0.0	-0.4
R (YLSH)	-0.5	0.1	0.5	-0.2	**0.9**	0.1	0.0	0.8	0.1	0.6	0.2	0.2	0.1	0.8	0.1	0.1
S (HTSH)	27.6	-0.1	25.2	34.0	26.8	5.9	-21.3	10.8	-21.7	28.9	-2.6	4.6	-21.7	1.6	-2.2	7.7
R (HTSH)	23.4	-0.3	**45.0**	24.0	-18.4	18.4	-23.1	17.3	-15.9	19.1	23.6	9.9	-11.6	15.0	2.9	31.0
S (BWT)	5.6	2.3	4.2	7.2	7.0	1.5	-2.3	-1.5	-0.6	2.1	2.1	1.6	-1.2	-0.2	2.6	-0.7
R (BWT)	3.4	0.7	1.0	**3.8**	-0.9	1.0	0.4	-4.0	-2.3	-2.3	0.7	2.5	-0.1	-2.8	3.4	1.4
S (NH)	0.7	0.1	0.2	2.6	0.5	0.7	-0.1	0.0	1.1	0.6	0.2	0.3	-0.3	-0.3	-0.1	-0.8
R (NH)	0.4	0.4	1.0	0.9	**1.2**	1.1	0.3	0.9	**1.2**	0.4	0.8	**1.2**	-0.4	0.5	0.7	-0.3
S (NF)	22.1	-1.8	19.7	37.2	17.5	7.0	-19.7	7.7	7.5	15.9	8.8	12.2	-13.4	9.2	3.6	-16.0
R (NF)	15.9	9.0	**35.8**	12.8	19.9	13.9	1.5	21.7	27.4	10.7	19.6	25.6	-3.1	16.3	13.5	2.0
S (FL)	1.6	-0.2	0.8	2.8	1.9	0.7	-1.1	-1.4	-1.5	0.4	0.5	1.0	-0.9	-0.2	1.2	0.2
R (FL)	**2.8**	0.3	-0.8	2.5	-1.3	-0.2	-0.2	-3.9	-2.0	-2.6	-0.5	1.6	1.3	-2.6	2.3	0.3
S (FC)	2.2	0.7	2.2	2.1	3.1	1.2	-1.2	-1.1	0.4	0.9	1.2	0.6	-0.7	0.3	1.4	0.9
R (FC)	0.8	0.0	-0.6	**1.2**	-1.8	-0.7	-0.4	-3.4	-2.8	-2.5	-0.8	0.1	-0.4	-3.0	0.6	0.8
S (FRD)	0.6	0.2	0.6	0.6	0.9	0.4	-0.4	0.0	0.2	0.6	0.5	0.3	-0.2	0.2	0.6	0.1
R (FRD)	0.2	0.0	-0.3	0.3	-0.7	-0.3	-0.2	-1.2	-1.0	-0.8	-0.4	-0.1	-0.2	-1.0	0.1	**0.4**
S (PLD)	0.6	0.2	0.6	0.6	0.9	0.3	-0.3	0.0	0.1	0.6	0.5	0.3	-0.1	0.2	0.6	0.1
R (PLD)	0.2	0.0	-0.3	0.3	-0.7	-0.3	-0.1	-1.2	-1.0	-0.9	-0.4	-0.1	-0.2	-1.0	0.1	**0.4**
S (PED)	0.00	0.00	0.02	0.00	0.02	0.03	-0.02	0.01	0.01	0.02	0.00	-0.03	-0.03	-0.02	-0.01	0.01
R (PED)	0.01	0.01	-0.01	0.01	0.00	0.02	-0.01	-0.01	0.01	0.04	0.02	0.01	0.00	0.00	0.01	0.00

S = Selection differential, R = Response to selection, bold values are the highest observations, C04 = 1201K-1x9128-3, C05 = 1201K-1 x cv. Rose, C08 = 1201K-1 x *malaccensis*, C10 = 1201K-1 x SH3217, C11 = 1201K-1 x SH3362, C12 = 1438K-1 x 5610S-1, C13 = 1438K-1 x cv. Rose, C16 = 1438K-1 x *malaccensis*, C22 = 5610S-1 x 2180K-6, C27 = 660K-1 x *malaccensis*, C31 = 917K-2 x 5610S-1, C33 = 917K-2 x 9128–3, C34 = 917K-2 x cv. Rose, C37 = 917K-2 x *malaccensis*, C61 = 917K-2 x SH3362 and MxC4 = Matooke (EAHB) x Calcutta 4

The best cross in terms of yield and fruit size was C10 (1201K-1xSH3217). Many hybrids from this cross had the highest bunch weight (R = 3.8) characterized by longer fruit fingers, big fruit circumference and the highest pulp content. However, the plants were very tall with big girth. Their maturity period was shorter (about 4.5 months on average) and comparable to hybrids from EAHBxCalcutta 4. Generally, crosses involving SH3217, SH3362 and 9128–3 as male parents produced hybrids that had good fruit filling characteristics although they varied in Black Sigatoka resistance and suckering behavior. For example, crosses involving 9128–3 generated hybrids that had the lowest INSL.

Hybrids from a cross between 5610S-1 and 2180K-6 produced the highest number of leaves scored at flowering (R = 2.1). They had the highest resistance to Black Sigatoka as reflected by INSL (R = 7.2%) despite the parents being susceptible. They were the shortest (R = -62.3 cm) with smaller plant girth. Their average maturity period was almost two months more than the average of all parental lines (R = 54.6 days) and the longest of all other hybrids. Due to long maturity period the number of standing leaves at harvest was very low because of normal leaf senescence. Despite producing many fruit fingers and slightly more hands per bunch, their average yield and size of fruits were lower than those of the parents. However, some exceptional lines such as 25031S-7 (diploid) had sizable bunch with relatively big fruits.

Crosses involving *M*. *acuminata* ssp. *malaccensis* 250 as male parent produced hybrids that were tall, slender, with bunches that had many fruit fingers poorly filled with pulp but some individual genotype exceptions were observed. The hybrids were resistant to Black Sigatoka and had the highest number of functional leaves at harvesting. Hybrids from cv. Rose were slender and shorter and were the highest in sucker production while other traits varied considerably.

Hybrids from different cross combinations had longer maturity period (128–185 days) than EAHB. On average EAHB mature within 90 days after flowering while the average maturity period for all parental lines was 130 days.

### Genetic diversity of GS training population

Out of the nineteen SSR markers, eighteen were used to delineate the structure of the study population, because marker mMaCIR164 produced ambiguous allele profiles across samples. From 18 loci, 195 alleles were scored and the number of alleles per locus ranged between 4 and 18 with an average of 10.8. Polymorphism information content (PIC) of the markers was high with an average of 0.87 (0.53–0.95) while the major allele frequency was on average 0.22 (0.1–0.45).

**Fig 6:**
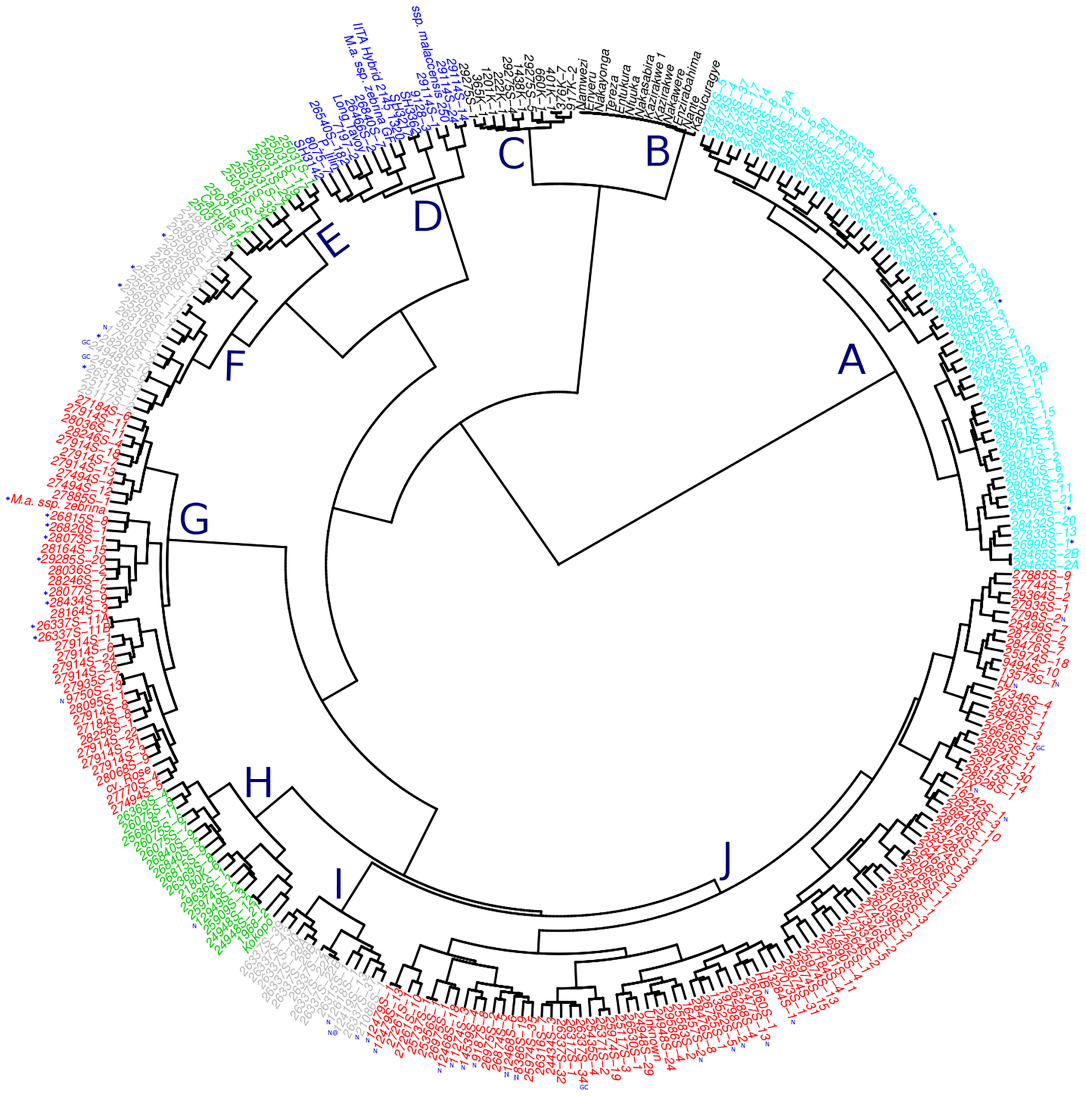
Dendrogram showing the genetic diversity of the genomic selection training population based on 19 informative SSR markers. The squared Euclidean distances were used to generate the hierarchical clusters based on ward.D criterion. Where cluster A = tetraploids (4x) by *M*. *a*. *spp*. *malaccensis* 250, * share only female parent, cluster B = matooke (EAHB), cluster C = tetraploids from EAHB (3x) by Calcutta 4 a wild diploid (2x), cluster D = wild and improved diploids, cluster E = Black Sigatoka resistant diploid hybrids, cluster F = hybrids of 5610S-1 as a male parent, * share grandparent Calcutta 4, GC = good for cooking and N = NARITA hybrid, cluster G = cv. Rose was the main male parent, * share genetic background, cluster H = Long Tavoy and Calcutta 4 are the grandparents, cluster I = mostly hybrids of SH3217 as male parent, N = NARITA, @ = released variety as NARITA 7/M9/Kiwangazi and cluster J = triploid hybrids with complex pedigree, most advanced hybrids such as NARITAs (N) are found in this cluster of which some are promising variety candidates and GC = good for cooking.

Despite the complex pedigree background of the GS population, SSR markers were informative enough to delineate the structure of the population (Fig 6). Hierarchical clustering based on Ward’s criterion revealed ten groups indicating that the genetic diversity of population was high. The triploid East African highland bananas clearly separated from other triploids. They had the lowest within group genetic diversity. The tetraploids that resulted from crossing EAHB by cv. ‘Calcutta 4’ and *M*. *acuminata* ssp. *malaccensis* 250 formed their own cluster but were closely linked to that of EAHB, thus supporting the hypothesis that the tetraploids were formed after fusion of unreduced gametes from triploid EAHB and haploid gametes from diploid cv. ‘Calcutta 4’ and *M*. *acuminata* ssp. *malaccensis* 250. The within cluster dispersion was rather homogenous and not highly diverse for the tetraploid hybrids probably due high allele dosage from EAHB. SSR data suggested that the tetraploid presumed to be hybrids of cv. Enzirabahima by *M*. *a malaccensis* 250 (29275S-1, 29275S-4 and 29275S-5), were in fact admixtures from pollination of EAHB with cv. ‘Calcutta 4’. These tetraploid inherited 17 alleles specific for cv. ‘Calcutta 4’ and none of ssp. *malaccensis* 250 specific alleles across the 18 SSR markers used.

Hierarchical clustering of hybrids was much influenced by male parents used in the cross. The biggest percentage of hybrids was produced from crosses involving tetraploids derived from EAHB and cv. ‘Calcutta 4’. Hybrids from ssp. *malaccensis* 250 were more distinct from the rest of the population and formed their own cluster. Four hybrids (26998S-1, 27074S-1, 28506S-1 and 27960s-1) presumed to be progeny of 2180K-6, cv. ‘Calcutta 4’ and cv. ‘Rose’ as male parents clustered together with ssp. *malaccensis* 250 hybrids. SSR genotype profiles suggested that those four hybrids were misidentified because they had ssp. *malaccensis* 250 specific alleles. The highest genetic diversity was observed in the diploid parents and between families. Diploids that were linked by pedigree clustered together but the within cluster differences were high compared to EAHB and tetraploids. Diploids such as cv. ‘Calcutta 4’, 861S-1, 5610S-1, 2180K-1, Kokopo, and cv. ‘Rose’ clustered with their hybrids. Hybrids derived from 5610S-1 x 2180K-1 were all diploids and closely related to cv. ‘Calcutta 4’ and 861S-1 and formed a separate cluster. Although the pedigree of 2180K-1 could not be traced, there is a possibility that one of its parents was cv. ‘Calcutta 4’. Hybrids from cv. ‘Long Tavoy’ and cv. ‘Calcutta 4’ were not easily delineated because of the close resemblance of these genotypes. One cluster (J) comprising of triploid hybrids showed high within cluster diversity. Majority of advanced hybrids especially NARITA hybrids comprising of potential candidate varieties are found in this cluster. The ssp. *zebrina* accessions included in the analysis clustered within the main clusters suggesting their genetic relatedness with other *acuminata* genotypes. In the population, some genotypes were duplicates. The duplicates identified included 28465S-2 (A&B), 26337S-11 (A&B) and 26337S-22 (A&B) while 27524S-12 (A&B) that were assumed to be duplicates were clarified to be genetically different although both were progeny of ssp. *malaccensis* 250. Other supposed unique genotypes were identified as likely clonal pairs, such as 24948S-9 and 24948S-10, 24948S-22 and 24948S-27, 25623S-11 and 25628S-11, 24948S-12 and 24948S-21, 12479S-1 and 12479S-13, 25737S-1 and 25356S-1, and 25066S-1 and 25066S-2.

## Discussion

### Trait evaluation

Bananas express many traits that are used to evaluate hybrids in breeding programs. These traits can be broadly classified as vegetative/agronomic (growth) traits, or yield and consumer appeal (fruit) traits. Growth and yield related traits are used to assess the level of introgression of resistance genes and this is done in the early evaluation trial. The index of non-spotted leaves (INSL) is a measure of resistance to Black Sigatoka, a fungal disease that causes rapid drying of leaves hence reducing the photosynthetic area [7]. Results from ANOVA obtained in this work showed that INSL was not significantly affected by cycle. However, the effect of level of input in field management on INSL depended on genotype. This suggests that resistance to Black Sigatoka might be under strong genetic control and less influenced by cycle.

Correlation analysis showed a positive correlation between INSL, NSLF and YLSF. However, these three had low but significant negative correlations with yield-related traits under low input field management conditions. These results suggest that whereas some Black Sigatoka resistant genotypes give good yield, others produce inferior fruits. Reduction in functional leaves and photosynthetic area has been shown to impact banana yield potential [7]. Tushemereirwe [36] indicated that Black Sigatoka reduced yield of EAHB by more than 30%. Our results show that under high input field management conditions, the impact of the disease on yield traits was less severe (Tables 1 and 2). This result is in agreement with Mobambo et al. [37] who reported that soil fertility had an effect on host plant response to Black Sigatoka and yield in plantains. The symptoms of Black Sigatoka often increase after flowering probably because at that time the ability of a plant to withstand the fungal attack is lowered as it commits most of the energy and resources to the developing inflorescence. Some genotypes had no functional leaves at harvest, indicating that they were very susceptible to Black Sigatoka after flowering. Selection of hybrids based on the number of functional leaves at harvest as a measure of resistance to Black Sigatoka should be done with caution because of the negative association between foliar symptoms to Black Sigatoka and fruit filling.

The present study shows that based on yield and growth traits, four groups of bananas existed in the training population that is, genotypes with high INSL and good fruit filling, high INSL with poor fruit filling, low INSL with good fruit filling and low INSL with poor fruit filling representing the four planes of the two components. However, PCA could not resolve the population structure into clear-cut clusters due to complex pedigrees, although Osuji et al. [38] used this approach to distinguish between different *Musa* triploids. This phenomenon could be attributed to differences in carbon source to sink capacities.

Plant physiological studies have shown that the balance between source and sink translocation of photosynthetic assimilates is key to plant productivity [39]. In bananas, Dens et al. [40] demonstrated the effect of manipulating the carbon source (C-source) and carbon sink (C-sink) of mother plant on ratoon crops in cv. ‘Williams’ and cv. ‘Grand Nain’ at a mat level. Their results showed genotype and environmental effect on flowering time, plant height and bunch size for the first ratoon crop. They concluded that the bunch was a larger C-sink than the ratoon crop. At individual plant level, it is likely that difference in C-source to C-sink capacity exists in bananas because our results showed that poor fruit filling genotypes were not significantly affected by cycle and field inputs. It can be postulated that when plants have a strong C-sink capacity they tend to have high yield with increased leaf senescence, while those with low C-sink capacity maintain many leaves with low yield at harvest. More physiological studies in banana are required to shed light on this aspect. It has been reported that at the time of flowering, the fruits and seeds became major sinks and any factor that reduces translocation of photosynthetic assimilates to fruits reduces the harvest index [41].

The training population consisted of poor and good fruit filling genotypes based on FL, FC, FRD and PLD. This characteristic was consistent across cycles and field management, with two overlapping peaks in a binary pattern (S1A Fig). However, given the consistence of the traits under different field conditions, there is likelihood that fruit filling is under control of one or few major-effect quantitative trait loci (QTL). Given that the training population was not a classical bi-parental mapping population this argument may not hold, but investigations using genome wide association studies while accounting for pedigree effect [42] may help to unravel the underlying genetic mechanisms using genome-wide markers such as SNPs.

This study did not find sufficient evidence to show that absolute apical dominance existed in our training population. Different levels of sucker regulation (1–25 suckers) were observed in different cross combinations. This result is in agreement with the observation made by Ortiz and Vuylsteke [43] that non-apical dominance genes were fixed in AA genotypes of *Musa*.

### GxE interaction

The effects of cycle and field input management on the genotype and how the genotype interacted with these two aspects of the environment were evaluated. The effect of cross combination was also assessed. Based on coefficients of determination and analysis of variance, genotype, cycle, field and their interactions had different levels of effect on trait variation among cross combinations and individual genotypes. While PHF and PG significantly increased at cycle 2, field management did not have a significant effect on these traits. This could be attributed to the fact that the suckers used were at different physiological maturity. Yield traits were also affected by cycle but the bi-modal distribution was maintained. When bananas are planted in the field they first undergo an establishment phase and build reserves that can accelerate growth of the daughter plants. Therefore, cycle 2 is best to compare genotypes especially with regard to yield traits. Tushemereirwe et al. [16] reported a cycle effect on traits when they analyzed some advanced hybrids, but it was not fully known whether this behavior occurred in different banana genotypes. The effect of cycle alone varied across traits depending on field management except for PED, HTSF and INSL that were most stable. It should however be noted that under optimum field management the cycle explains a small proportion of trait variation in genotypes because most traits had coefficient of determination values below 0.4 in GS2.

The present results show that different banana traits may have different genetic architecture with some traits influenced by GxE. In marker assisted selection this can hamper deployment of classical marker technologies that rely on identifying QTLs. Approaches such as genomic selection that utilize genome-wide markers in complex populations such as in this study provide an opportunity to dissect such traits and could be exploited by banana breeders to increase genetic gain per unit time. Genotype by environment interaction has been shown to affect the accuracy of genomic selection models [24, 44]. Therefore, understanding genotype trait variation across different environments is paramount.

Many hybrids generated from crossbreeding usually have inferior fruit size irrespective of the ploidy level. Such inferiority has been attributed to linkage drag from wild diploids [45]. Bananas have a long selection cycle, they are labor intensive, costly and require large land area for evaluation. Any technology that can discriminate the inferior genotypes from the good ones at a nursery stage could save a lot of resources and time for the breeders thus increasing the breeding efficiency. With the availability of the *Musa* reference genome [46, 47] and decreasing costs of next generation sequencing technologies, high density marker technologies such as genotyping by sequencing are available for many plant species [48]. This provides an opportunity to investigate the application of genomic selection in banana breeding.

### Performance of cross combinations

The true breeding value of a genotype is determined by the quality of hybrids produced when it is involved in a cross. By comparing the responses to selection (R) and selection differentials (S) of sixteen cross combinations it was concluded that no single cross combination presented all the good qualities targeted by the breeders in hybrids. This further explains the complex trait variation observed within study population. No attempt was made to determine heritability of the traits because of unbalanced design and the possibility of confounding from heterosis [31]. Some hybrids that had many active leaves at harvest showed variation in fruit filling. Performance of the hybrids was greatly influenced by the male parent involved in the cross. Although both diploids and tetraploids had 50% segregation opportunity, the tetraploids were genetically very similar, whereas the diploids were more genetically diverse with the exception of SH3217 and SH3362 that were closely related. Crosses involving diploid SH3217, SH3362 and 9128–3 produced hybrids which were superior in yield compared to other crosses. These diploids are parthenocarpic, with big fruits and many hands (clusters) per bunch. The best cross combination was C10 (120K-1 x SH3217) that produced hybrids that were fairly resistant to Black Sigatoka, high yielding and quick maturing. Despite the susceptibility of 1201K-1 parent to Black Sigatoka, segregation was observed and some hybrids that had some acceptable levels of resistance were produced.

Tenkouano et al. [49] reported a 4-fold contribution of male parents toward yield traits while Rowe and Rosales [50] highlighted that breeding for improved diploids with pest and disease resistance, parthenocarpy and good yield was the best strategy in banana improvement. Gene pyramiding has also been suggested so that multiple introgressions of good traits are possible [51]. Most of the improved varieties produced by crossbreeding are triploid and all assumed to be completely sterile but no research has been conducted to evaluate their fertility. Further improvement of these triploids is necessary given that no single hybrid has all traits desired by farmers and consumers. The 2x by 2x hybrids were all diploid and some had sizable bunches compared to other diploids in the core breeding set, i.e. could be interesting as improved 2x parents. Further evaluation of these diploids for pollen viability and parthenocarpy will be necessary before they are incorporated in the core breeding set despite their long maturity period. Hybrids that take four months to mature may be considered quick maturing, given that the majority take more than four months.

### Genetic diversity of GS training population

Whereas principal component analysis on cross combinations and individual genotypes showed that high genetic diversity existed in the training population, its power to resolve the structure of the population into clear-cut clusters that make biological sense was limited. This was attributed to complex pedigrees in the population with 77 cross combinations represented. The half-sib families were closely related to one another with which they shared a common parent. The population was interconnected due to shared parents in their pedigree. Use of SSR markers proved valuable in delineating the population structure that could be easily interpreted. The set of markers used was reported to be informative and has been used on genotyping the banana collection from the International Transit Center [32]. The polymorphism information content (PIC) of 0.87 was high enough to resolve even the closest genotypes. Up to ten unique clusters were resolved and results showed that clustering was mostly influenced by the genetic diversity in diploid parents.

Triploid EAHB and tetraploids derived from them by crossing with cv. ‘Calcutta 4’ formed two distinct but closely related clusters, supporting the hypothesis of production of unreduced 3n and reduced n gametes during meiotic events in the tetraploid progenitors [52]. Despite the high PIC of the markers, the EAHB showed a very low genetic diversity consistent with the hypothesis that this group of bananas is an ancient clone set [9]. Even with a high number of polymorphic SSR markers Kitavi et al. and Karamura et al. [9, 53] failed to separate this group into the corresponding phenotype-based clone sets of Karamura [1]. However, some genetic differences were observed between some individual genotypes that could be attributed to mutations within this ancient clone set. The population was predominated with genetic introgression from cv. ‘Calcutta 4’. Hybrids from *M*. *acuminata* ssp. *malaccensis* 250 formed a distinct cluster. Three tetraploids presumed to be arising from a cross of EAHB with ssp. *malaccensis* 250 grouped together with those derived from EAHB by cv. ‘Calcutta 4’. The presence of Calcutta 4-specific alleles in these tetraploids and the absence of ssp. *malaccensis* 250 specific alleles suggest that these hybrids are progeny of EAHB by cv. ‘Calcutta 4’ hence the high genetic relationship with the rest of the tetraploids. Nevertheless, these tetraploids should be tested as parents to determine their breeding values so that the breeding genetic pool is expanded.

The SSR markers proved useful in identifying duplicates and closely related genotypes based on pedigree background. A combination of highly polymorphic SSR markers and the power of Ward’s clustering method that minimizes the within-group dispersion [34] in the Euclidean space helped to resolve the structure of the population that was highly interlinked by pedigree background. The high level of genetic complexity observed in this population represents different recombination events that make it suitable as a training population for genomic selection.

Apart from obtaining important data on the banana GS training population, important lessons were learned during the course of this work. Dedicated efforts are required to understand the genome organization of bananas through cytological approaches. Ploidy analysis should be routinely employed in breeding programs to differentiate ploidy levels so that different selection criteria are used to select hybrids intended for the breeding pipeline from those eligible for variety release. Despite a majority of the improved hybrids being triploids, their fertility should be tested so that further improvements can be made on them as a way to achieve gene pyramiding while minimizing inbreeding.

## Conclusion

The response of genotype trait expression to cycle and field management practices varied greatly. The largest proportion of genetic variation was due to the greater genetic diversity of male parents used in crosses since the tetraploids used in the majority of crosses as female parents were genetically related. Yield traits accounted for 31–35% of the total principal component variation observed in the population and were loaded on the first component while vegetative traits contributed to the second component with 15–22%. A high level of correlation within vegetative- and yield-related traits was observed but correlation between vegetative and yield traits was low and depended on the interaction with field management practices. Therefore, genomic selection models could be developed for traits that are easy to measure. It is likely that the predictive ability of traits that are difficult to phenotype will be similar to traits easily measured but highly correlated. The study population was observed to be genetically diverse with complex pedigree structure. Yield-related traits showed a bi-modal distribution, which was not influenced by cycle or field management. Resistance to Black Sigatoka was also stable across cycles but varied under different field management depending on the genotype. Principal component analysis could not delineate this complex population structure but the application of SSR markers in combination with Ward’s hierarchical clustering proved powerful and resolved the structure into biologically meaningful groups.

## Supporting information

S1 Fig Variation in fruit characteristics. (A) is a histogram showing the bimodal distribution of fruit circumference (FC), (B) cross sections of poor filling fruits, (C) good filling fruits with fruit diameter (FRD) and pulp diameter (PLD) values in cm, and (D) poor filling and good filling banana fruits.
(TIF)

S1 Table List of genotypes in a banana genomic selection training population. (DOCX)

S2 Table Data used to calculate selection differential and response to selection for the sixteen cross combinations. (XLSX)

S3 Table Summary of all trait variations in response to cycle and field management. (DOCX)
