# Supplementary figures and images for: Trait variation and genetic diversity in a banana genomic selection training population
Source: PLoS One. 2017 Jun 6;12(6):e0178734. doi: 10.1371/journal.pone.0178734 (PMC5460855; doi:10.1371/journal.pone.0178734)

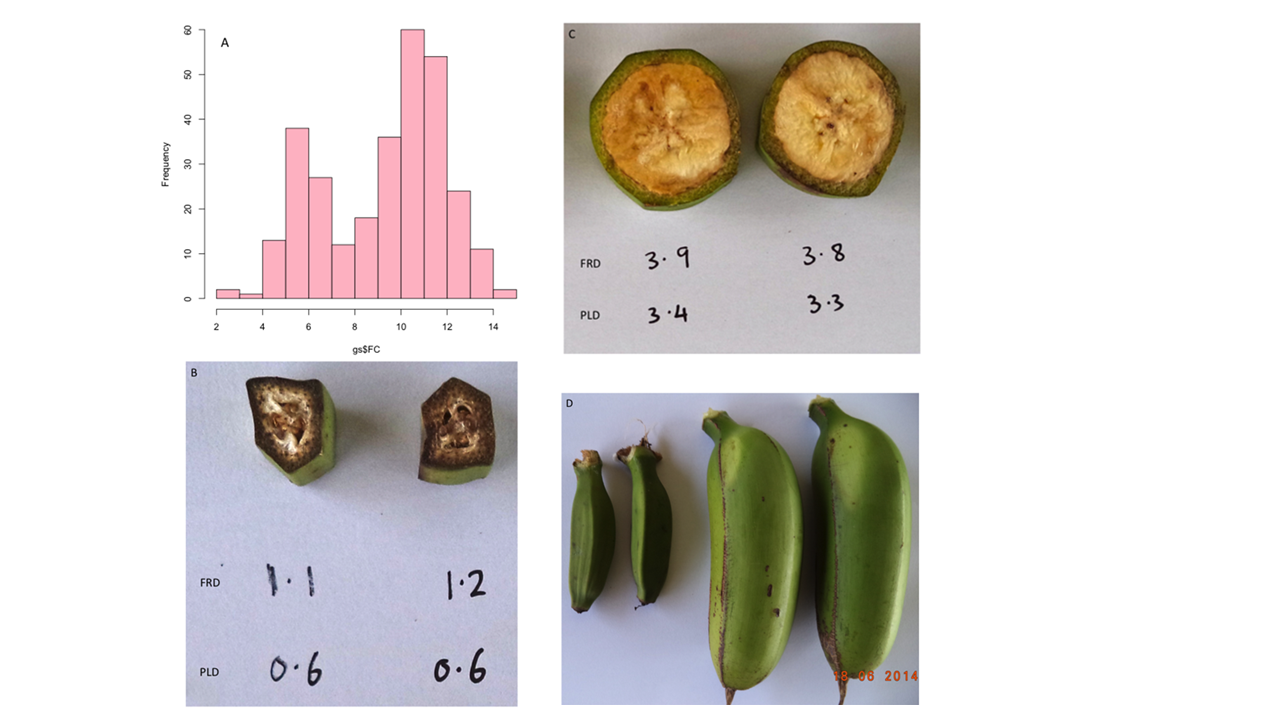

Supplement: S1 Fig — (A) is a histogram showing the bimodal distribution of fruit circumference (FC), (B) cross sections of poor filling fruits, (C) good filling fruits with fruit diameter (FRD) and pulp diameter (PLD) values in cm, and (D) poor filling and good filling banana fruits. (TIF) [file pone.0178734.s001.tif]
