# Supplementary material for: Trait variation and genetic diversity in a banana genomic selection training population
Source: PLoS One. 2017 Jun 6;12(6):e0178734. doi: 10.1371/journal.pone.0178734 (PMC5460855; doi:10.1371/journal.pone.0178734)
Supplement: S1 Table — (DOCX) [file pone.0178734.s002.docx]

**S1 Table. List of genotypes in the genomic selection training population**

| S/No | Cross | Genotype name | Female parent | Male parent | Ploidy | Description |
| --- | --- | --- | --- | --- | --- | --- |
| 1 |  | Enzirabahima |  |  | 3x | Parent |
| 2 |  | Kabucuragye |  |  | 3x | Parent |
| 3 |  | Tereza |  |  | 3x | Parent |
| 4 |  | Enyeru |  |  | 3x | Parent |
| 5 |  | Nakayonga |  |  | 3x | Parent |
| 6 |  | Namwezi |  |  | 3x | Parent |
| 7 |  | Entukura |  |  | 3x | Parent |
| 8 |  | Nakasabira |  |  | 3x | Parent |
| 9 |  | Nakawere |  |  | 3x | Parent |
| 10 |  | Nante |  |  | 3x | Parent |
| 11 |  | Kazirakwe |  |  | 3x | Parent |
| 12 |  | Nfuuka |  |  | 3x | Parent |
| 13 |  | Calcutta 4 |  |  | 2x | Parent |
| 14 | C45 | 1201K-1 | Nakawere | Calcutta 4 | 4x | Parent |
| 15 | C41 | 917K-2 | Enzirabahima | Calcutta 4 | 4x | Parent |
| 16 | C41 | 660K-1 | Enzirabahima | Calcutta 4 | 4x | Parent |
| 17 | C40 | 1438K-1 | Entukura | Calcutta 4 | 4x | Parent |
| 18 | C51 | 222K-1 | Nfuuka | Calcutta 4 | 4x | Parent |
| 19 | C49 | 376K-7 | Nante | Calcutta 4 | 4x | Parent |
| 20 | C67 | 365K-1 | Kabucuragye | Calcutta 4 | 4x | Parent |
| 21 | C40 | 401K-1 | Entukura | Calcutta 4 | 4x | Parent |
| 22 | C66 | 2180K-6 |  |  | 2x | Parent |
| 23 | C53 | 8075-7 | SH3362 | Calcutta 4 | 2x | Parent |
| 24 | C54 | 7197-2 | SH3362 | Long Tavoy | 2x | Parent |
| 25 | C63 | SH3142 | SH1734 | Pisang Jari Buaya | 2x | Parent |
| 26 | C64 | SH3362 | SH3217 | SH3142 | 2x | Parent |
| 27 | C52 | SH3217 | SH2095 | SH2766 | 2x | Parent |
| 28 | C43 | 5610S-1 | Kabucuragye | 7197-2 | 2x | Parent |
| 29 | C65 | 9128-3 | Tjau lagada | Pisang lilin | 2x | Parent |
| 30 | C57 | 1968-2 | Who-gu | Calcutta 4 | 3x | Parent |
| 31 | C48 | 861S-1 | Namwezi | Calcutta 4 | 2x | Parent |
| 32 |  | cv. Rose |  |  | 2x | Parent |
| 33 |  | Pisang Lilin |  |  | 2x | Parent |
| 34 |  | Kokopo |  |  | 2x | Parent |
| 35 |  | Long Tavoy |  |  | 2x | Parent |
| 36 |  | *malaccensis* 250 |  |  | 2x | Parent |
| 37 | C01 | 28165S-1 | 1201K-1 | 1968-2 | 3x | Hybrid |
| 38 | C02 | 25583S-2 | 1201K-1 | 5610S-1 | 3x | Hybrid |
| 39 | C02 | 26660S-1 | 1201K-1 | 5610S-1 | 3x | Hybrid |
| 40 | C02 | 28434S-9 | 1201K-1 | 5610S-1 | 3x | Hybrid |
| 41 | C68 | 17503S-3 | 1201K-1 | 7197-2 | 3x | Hybrid |
| 42 | C03 | 16242S-1 | 1201K-1 | 8075-7 | 3x | Hybrid |
| 43 | C04 | 12479S-1 | 1201K-1 | 9128-3 | 3x | Hybrid |
| 44 | C04 | 12479S-13 | 1201K-1 | 9128-3 | 3x | Hybrid |
| 45 | C04 | 26317S-1 | 1201K-1 | 9128-3 | 3x | Hybrid |
| 46 | C04 | 27262S-1 | 1201K-1 | 9128-3 | 3x | Hybrid |
| 47 | C04 | 27262S-3 | 1201K-1 | 9128-3 | 3x | Hybrid |
| 48 | C05 | 27770S-20 | 1201K-1 | cv. Rose | 3x | Hybrid |
| 49 | C05 | 27770S-4 | 1201K-1 | cv. Rose | 3x | Hybrid |
| 50 | C05 | 27935S-1 | 1201K-1 | cv. Rose | 3x | Hybrid |
| 51 | C05 | 27960S-1 | 1201K-1 | cv. Rose | 3x | Hybrid |
| 52 | C05 | 28036S-11 | 1201K-1 | cv. Rose | 3x | Hybrid |
| 53 | C05 | 28036S-2 | 1201K-1 | cv. Rose | 3x | Hybrid |
| 54 | C05 | 28164S-3 | 1201K-1 | cv. Rose | 3x | Hybrid |
| 55 | C05 | 28246S-4 | 1201K-1 | cv. Rose | 3x | Hybrid |
| 56 | C05 | 28246S-7 | 1201K-1 | cv. Rose | 3x | Hybrid |
| 57 | C05 | 27935S-7 | 1201K-1 | cv. Rose | 3x | Hybrid |
| 58 | C06 | 26363S-1 | 1201K-1 | Kokopo | 3x | Hybrid |
| 59 | C07 | 26075S-6 | 1201K-1 | Long Tavoy | 3x | Hybrid |
| 60 | C07 | 26075S-7 | 1201K-1 | Long Tavoy | 3x | Hybrid |
| 61 | C07 | 26075S-8 | 1201K-1 | Long Tavoy | 3x | Hybrid |
| 62 | C08 | 27346S-2 | 1201K-1 | *malaccensis* 250 | 3x | Hybrid |
| 63 | C08 | 27346S-4 | 1201K-1 | *malaccensis* 250 | 3x | Hybrid |
| 64 | C08 | 27437S-1 | 1201K-1 | *malaccensis* 250 | 3x | Hybrid |
| 65 | C08 | 27579S-1 | 1201K-1 | *malaccensis* 250 | 3x | Hybrid |
| 66 | C08 | 27579S-3 | 1201K-1 | *malaccensis* 250 | 3x | Hybrid |
| 67 | C08 | 28030S-2 | 1201K-1 | *malaccensis* 250 | 3x | Hybrid |
| 68 | C08 | 28030S-6 | 1201K-1 | *malaccensis* 250 | 3x | Hybrid |
| 69 | C08 | 28071S-1 | 1201K-1 | *malaccensis* 250 | 3x | Hybrid |
| 70 | C08 | 28465S-2 | 1201K-1 | *malaccensis* 250 | 3x | Hybrid |
| 71 | C08 | 28465S-21 | 1201K-1 | *malaccensis* 250 | 3x | Hybrid |
| 72 | C08 | 28479S-2 | 1201K-1 | *malaccensis* 250 | 3x | Hybrid |
| 73 | C10 | 26337S-22A | 1201K-1 | SH3217 | 3x | Hybrid |
| 74 | C10 | 26337S-40 | 1201K-1 | SH3217 | 3x | Hybrid |
| 75 | C11 | 26840S-7 | 1201K-1 | SH3362 | 2x | Hybrid |
| 76 | C09 | 26315S-1 | 1201K-1 | SH3142 | 3x | Hybrid |
| 77 | C10 | 12419S-13 | 1201K-1 | SH3217 | 3x | Hybrid |
| 78 | C10 | 26337S-11A | 1201K-1 | SH3217 | 3x | Hybrid |
| 79 | C10 | 26337S-2 | 1201K-1 | SH3217 | 3x | Hybrid |
| 80 | C10 | 26337S-34 | 1201K-1 | SH3217 | 3x | Hybrid |
| 81 | C10 | 26337S-37 | 1201K-1 | SH3217 | 3x | Hybrid |
| 82 | C10 | 26337S-39 | 1201K-1 | SH3217 | 3x | Hybrid |
| 83 | C10 | 26337S-43 | 1201K-1 | SH3217 | 3x | Hybrid |
| 84 | C10 | 28263S-2 | 1201K-1 | SH3217 | 3x | Hybrid |
| 85 | C11 | 12618S-1 | 1201K-1 | SH3362 | 3x | Hybrid |
| 86 | C11 | 26316S-7 | 1201K-1 | SH3362 | 3x | Hybrid |
| 87 | C11 | 26840S-10 | 1201K-1 | SH3362 | 3x | Hybrid |
| 88 | C58 | 25328S-3 | 1438K-1 | 1537K-1 | 3x | Hybrid |
| 89 | C12 | 24948S-10 | 1438K-1 | 5610S-1 | 3x | Hybrid |
| 90 | C12 | 24948S-13 | 1438K-1 | 5610S-1 | 3x | Hybrid |
| 91 | C12 | 24948S-24 | 1438K-1 | 5610S-1 | 3x | Hybrid |
| 92 | C12 | 24948S-9 | 1438K-1 | 5610S-1 | 3x | Hybrid |
| 93 | C69 | 26060S-1 | 1438K-1 | 9128-3 | 3x | Hybrid |
| 94 | C70 | 13573S-1 | 1438K-1 | 9719-7 | 3x | Hybrid |
| 95 | C13 | 27914S-1 | 1438K-1 | cv. Rose | 3x | Hybrid |
| 96 | C13 | 27914S-13 | 1438K-1 | cv. Rose | 3x | Hybrid |
| 97 | C13 | 28095S-1 | 1438K-1 | cv. Rose | 3x | Hybrid |
| 98 | C13 | 27264S-2 | 1438K-1 | cv. Rose | 2x | Hybrid |
| 99 | C13 | 27914S-24 | 1438K-1 | cv. Rose | 3x | Hybrid |
| 100 | C13 | 27914S-26 | 1438K-1 | cv. Rose | 3x | Hybrid |
| 101 | C13 | 27914S-3 | 1438K-1 | cv. Rose | 3x | Hybrid |
| 102 | C14 | 25474S-1 | 1438K-1 | Kokopo | 3x | Hybrid |
| 103 | C15 | 26369S-4 | 1438K-1 | Long Tavoy | 3x | Hybrid |
| 104 | C16 | 28481S-1 | 1438K-1 | *malaccensis* 250 | 3x | Hybrid |
| 105 | C16 | 28561S-2 | 1438K-1 | *malaccensis* 250 | 3x | Hybrid |
| 106 | C19 | 26725S-1 | 1438K-1 | SH3362 | 3x | Hybrid |
| 107 | C17 | 25499S-7 | 1438K-1 | SH3142 | 3x | Hybrid |
| 108 | C18 | 26039S-2 | 1438K-1 | SH3217 | 3x | Hybrid |
| 109 | C20 | 26466S-2 | 1977K-1 | 5610S-1 | 3x | Hybrid |
| 110 | C20 | 26466S-5 | 1977K-1 | 5610S-1 | 3x | Hybrid |
| 111 | C71 | 22598S-2 | 365K-1 | 1201K-1 | 3x | Hybrid |
| 112 | C59 | 14539S-4 | 365K-1 | 660K-1 | 3x | Hybrid |
| 113 | C21 | 9750S-13 | 401K-1 | 9128-3 | 3x | Hybrid |
| 114 | C22 | 25031S-1 | 5610S-1 | 2180K-6 | 2x | Hybrid |
| 115 | C22 | 25031S-15 | 5610S-1 | 2180K-6 | 2x | Hybrid |
| 116 | C22 | 25031S-16 | 5610S-1 | 2180K-6 | 2x | Hybrid |
| 117 | C22 | 25031S-17 | 5610S-1 | 2180K-6 | 2x | Hybrid |
| 118 | C22 | 25031S-19 | 5610S-1 | 2180K-6 | 2x | Hybrid |
| 119 | C22 | 25031S-27 | 5610S-1 | 2180K-6 | 2x | Hybrid |
| 120 | C22 | 25031S-33 | 5610S-1 | 2180K-6 | 2x | Hybrid |
| 121 | C22 | 25031S-34 | 5610S-1 | 2180K-6 | 2x | Hybrid |
| 122 | C22 | 25031S-7 | 5610S-1 | 2180K-6 | 2x | Hybrid |
| 123 | C24 | 24583S-2 | 660K-1 | 5610S-1 | 3x | Hybrid |
| 124 | C24 | 26260S-3 | 660K-1 | 5610S-1 | 3x | Hybrid |
| 125 | C25 | 13284S-1 | 660K-1 | 9128-3 | 3x | Hybrid |
| 126 | C25 | 25371S-2 | 660K-1 | 9128-3 | 3x | Hybrid |
| 127 | C25 | 9187S-8 | 660K-1 | 9128-3 | 3x | Hybrid |
| 128 | C26 | 26709S-1 | 660K-1 | Calcutta 4 | 3x | Hybrid |
| 129 | C27 | 27713S-1 | 660K-1 | *malaccensis* 250 | 3x | Hybrid |
| 130 | C27 | 27825S-4 | 660K-1 | *malaccensis* 250 | 3x | Hybrid |
| 131 | C27 | 27873S-18 | 660K-1 | *malaccensis* 250 | 3x | Hybrid |
| 132 | C27 | 27873S-38 | 660K-1 | *malaccensis* 250 | 3x | Hybrid |
| 133 | C27 | 27873S-4 | 660K-1 | *malaccensis* 250 | 3x | Hybrid |
| 134 | C27 | 27873S-5 | 660K-1 | *malaccensis* 250 | 3x | Hybrid |
| 135 | C27 | 28188S-2 | 660K-1 | *malaccensis* 250 | 3x | Hybrid |
| 136 | C28 | 25623S-11 | 8817S-1 | 917K-2 | 3x | Hybrid |
| 137 | C29 | 28492S-1 | 917K-2 | 1968-2 | 3x | Hybrid |
| 138 | C30 | 26998S-1 | 917K-2 | 2180K-6 | 3x | Hybrid |
| 139 | C30 | 27074S-1 | 917K-2 | 2180K-6 | 3x | Hybrid |
| 140 | C31 | 25117S-1 | 917K-2 | 5610S-1 | 3x | Hybrid |
| 141 | C31 | 25117S-2 | 917K-2 | 5610S-1 | 3x | Hybrid |
| 142 | C31 | 25117S-3 | 917K-2 | 5610S-1 | 3x | Hybrid |
| 143 | C31 | 25508S-1 | 917K-2 | 5610S-1 | 3x | Hybrid |
| 144 | C31 | 25628S-11 | 917K-2 | 5610S-1 | 3x | Hybrid |
| 145 | C31 | 26815S-3 | 917K-2 | 5610S-1 | 3x | Hybrid |
| 146 | C31 | 26815S-8 | 917K-2 | 5610S-1 | 3x | Hybrid |
| 147 | C31 | 26815S-9 | 917K-2 | 5610S-1 | 3x | Hybrid |
| 148 | C31 | 26990S-10 | 917K-2 | 5610S-1 | 3x | Hybrid |
| 149 | C31 | 26990S-11 | 917K-2 | 5610S-1 | 3x | Hybrid |
| 150 | C31 | 26990S-4 | 917K-2 | 5610S-1 | 3x | Hybrid |
| 151 | C31 | 27073S-1 | 917K-2 | 5610S-1 | 3x | Hybrid |
| 152 | C31 | 27744S-1 | 917K-2 | 5610S-1 | 3x | Hybrid |
| 153 | C60 | 12949S-2 | 917K-2 | 7197-2 | 3x | Hybrid |
| 154 | C60 | 25909S-3 | 917K-2 | 7197-2 | 3x | Hybrid |
| 155 | C32 | 25089S-4 | 917K-2 | 861S-1 | 3x | Hybrid |
| 156 | C33 | 19798S-2 | 917K-2 | 9128-3 | 3x | Hybrid |
| 157 | C33 | 24434S-3 | 917K-2 | 9128-3 | 3x | Hybrid |
| 158 | C33 | 25435S-11 | 917K-2 | 9128-3 | 3x | Hybrid |
| 159 | C33 | 25435S-4 | 917K-2 | 9128-3 | 3x | Hybrid |
| 160 | C33 | 25737S-1 | 917K-2 | 9128-3 | 3x | Hybrid |
| 161 | C33 | 26288S-4 | 917K-2 | 9128-3 | 3x | Hybrid |
| 162 | C33 | 26975S-1 | 917K-2 | 9128-3 | 3x | Hybrid |
| 163 | C33 | 26975S-2 | 917K-2 | 9128-3 | 3x | Hybrid |
| 164 | C33 | 7798S-2 | 917K-2 | 9128-3 | 3x | Hybrid |
| 165 | C34 | 27184S-4 | 917K-2 | cv. Rose | 3x | Hybrid |
| 166 | C34 | 27885S-9 | 917K-2 | cv. Rose | 3x | Hybrid |
| 167 | C34 | 27184S-8 | 917K-2 | cv. Rose | 3x | Hybrid |
| 168 | C34 | 27494S-12 | 917K-2 | cv. Rose | 3x | Hybrid |
| 169 | C34 | 27494S-4 | 917K-2 | cv. Rose | 3x | Hybrid |
| 170 | C34 | 27494S-5 | 917K-2 | cv. Rose | 3x | Hybrid |
| 171 | C34 | 28068S-9 | 917K-2 | cv. Rose | 3x | Hybrid |
| 172 | C34 | 27184S-6 | 917K-2 | cv. Rose | 3x | Hybrid |
| 173 | C34 | 27885S-1 | 917K-2 | cv. Rose | 3x | Hybrid |
| 174 | C35 | 24410S-2 | 917K-2 | Kokopo | 3x | Hybrid |
| 175 | C36 | 25680S-11 | 917K-2 | Long Tavoy | 3x | Hybrid |
| 176 | C36 | 25680S-13 | 917K-2 | Long Tavoy | 3x | Hybrid |
| 177 | C37 | 27261S-1 | 917K-2 | *malaccensis* 250 | 3x | Hybrid |
| 178 | C37 | 27261S-10 | 917K-2 | *malaccensis* 250 | 3x | Hybrid |
| 179 | C37 | 27261S-11 | 917K-2 | *malaccensis* 250 | 3x | Hybrid |
| 180 | C37 | 27334S-5 | 917K-2 | *malaccensis* 250 | 3x | Hybrid |
| 181 | C37 | 27401S-1 | 917K-2 | *malaccensis* 250 | 3x | Hybrid |
| 182 | C37 | 27524S-12A | 917K-2 | *malaccensis* 250 | 3x | Hybrid |
| 183 | C37 | 27524S-12B | 917K-2 | *malaccensis* 250 | 3x | Hybrid |
| 184 | C37 | 27524S-22 | 917K-2 | *malaccensis* 250 | 3x | Hybrid |
| 185 | C37 | 27524S-30 | 917K-2 | *malaccensis* 250 | 3x | Hybrid |
| 186 | C37 | 27833S-10 | 917K-2 | *malaccensis* 250 | 3x | Hybrid |
| 187 | C37 | 27833S-13 | 917K-2 | *malaccensis* 250 | 3x | Hybrid |
| 188 | C37 | 27886S-5 | 917K-2 | *malaccensis* 250 | 3x | Hybrid |
| 189 | C37 | 28033S-14 | 917K-2 | *malaccensis* 250 | 3x | Hybrid |
| 190 | C37 | 28033S-15 | 917K-2 | *malaccensis* 250 | 3x | Hybrid |
| 191 | C37 | 28033S-18 | 917K-2 | *malaccensis* 250 | 3x | Hybrid |
| 192 | C37 | 28033S-23 | 917K-2 | *malaccensis* 250 | 3x | Hybrid |
| 193 | C37 | 28033S-3 | 917K-2 | *malaccensis* 250 | 3x | Hybrid |
| 194 | C37 | 28060S-8 | 917K-2 | *malaccensis* 250 | 3x | Hybrid |
| 195 | C37 | 28200S-3 | 917K-2 | *malaccensis* 250 | 3x | Hybrid |
| 196 | C37 | 28257S-1 | 917K-2 | *malaccensis* 250 | 3x | Hybrid |
| 197 | C37 | 28257S-2 | 917K-2 | *malaccensis* 250 | 3x | Hybrid |
| 198 | C37 | 28257S-4 | 917K-2 | *malaccensis* 250 | 3x | Hybrid |
| 199 | C37 | 28432S-19 | 917K-2 | *malaccensis* 250 | 3x | Hybrid |
| 200 | C37 | 28432S-20 | 917K-2 | *malaccensis* 250 | 3x | Hybrid |
| 201 | C37 | 28432S-3 | 917K-2 | *malaccensis* 250 | 3x | Hybrid |
| 202 | C37 | 28780S-1 | 917K-2 | *malaccensis* 250 | 3x | Hybrid |
| 203 | C61 | 26874S-5 | 917K-2 | SH3362 | 3x | Hybrid |
| 204 | C38 | 12468S-18 | 917K-2 | SH3217 | 3x | Hybrid |
| 205 | C38 | 12477S-13 | 917K-2 | SH3217 | 3x | Hybrid |
| 206 | C38 | 8386S-19 | 917K-2 | SH3217 | 3x | Hybrid |
| 207 | C61 | 13522S-5 | 917K-2 | SH3362 | 3x | Hybrid |
| 208 | C61 | 25974S-? | 917K-2 | SH3362 | 3x | Hybrid |
| 209 | C61 | 25974S-19 | 917K-2 | SH3362 | 3x | Hybrid |
| 210 | C61 | 25974S-21 | 917K-2 | SH3362 | 3x | Hybrid |
| 211 | C61 | 25974S-30 | 917K-2 | SH3362 | 3x | Hybrid |
| 212 | C61 | 25974S-35 | 917K-2 | SH3362 | 3x | Hybrid |
| 213 | C61 | 26666S-1 | 917K-2 | SH3362 | 3x | Hybrid |
| 214 | C61 | 28476S-7 | 917K-2 | SH3362 | 3x | Hybrid |
| 215 | C61 | 9494S-10 | 917K-2 | SH3362 | 3x | Hybrid |
| 216 | C62 | 16457S-2 | Entukura | 365K-1 | 3x | Hybrid |
| 217 | C39 | 26540S-182 | Entukura | 8075-7 | 2x | Hybrid |
| 218 | C41 | 28260S-2 | Enzirabahima | Calcutta 4 | 3x | Hybrid |
| 219 | C72 | 21086S-1 | Kazirakwe | 7197-2 | 3x | Hybrid |
| 220 | C46 | 28073S-1 | Namwezi | 7197-2 | 3x | Hybrid |
| 221 | C55 | 25356S-1 | Tereza | 7197-2 | 3x | Hybrid |
| 222 | C75 | HB | unknown | unknown | 3x | Hybrid |
| 223 | C76 | HJ | unknown | unknown | 3x | Hybrid |
| 224 | C77 | HX | unknown | unknown | 3x | Hybrid |
| 225 | C10 | 26337S-11B | 1201K-1 | SH3217 | 3x | Hybrid |
| 226 | C73 | 16285S-13 | Calcutta 4 | 660K-1 | 2x | Hybrid |
| 227 | C10 | 26337S-22B | 1201K-1 | SH3217 | 3x | Hybrid |
| 228 | C73 | 16285S-3 | Calcutta 4 | 660K-1 | 2x | Hybrid |
| 229 | C10 | 26337S-28 | 1201K-1 | SH3217 | 3x | Hybrid |
| 230 | C14 | 25066S-1 | 1438K-1 | Kokopo | 3x | Hybrid |
| 231 | C73 | 16285S-6 | Calcutta 4 | 660K-1 | 2x | Hybrid |
| 232 | C14 | 25066S-2 | 1438K-1 | Kokopo | 3x | Hybrid |
| 233 | C73 | 16285S-8 | Calcutta 4 | 660K-1 | 2x | Hybrid |
| 234 | C61 | 25974S-11 | 917K-2 | SH3362 | 3x | Hybrid |
| 235 | C61 | 25974S-15 | 917K-2 | SH3362 | 3x | Hybrid |
| 236 | C14 | 25457S-1 | 1438K-1 | Kokopo | 3x | Hybrid |
| 237 | C74 | 16191S-6 | Calcutta 4 | 917K-2 | 2x | Hybrid |
| 238 | C35 | 24797S-7 | 917K-2 | Kokopo | 3x | Hybrid |
| 239 | C35 | 25102S-1 | 917K-2 | Kokopo | 3x | Hybrid |
| 240 | C44 | 28452S-11 | Nakasabira | Calcutta 4 | 3x | Hybrid |
| 241 | C37 | 28033S-9 | 917K-2 | *malaccensis* 250 | 3x | Hybrid |
| 242 | C61 | 25974S-13 | 917K-2 | SH3362 | 3x | Hybrid |
| 243 | C34 | 28256S-1 | 917K-2 | cv. Rose | 3x | Hybrid |
| 244 | C61 | 25974S-17 | 917K-2 | SH3362 | 4x | Hybrid |
| 245 | C38 | 12468S-6 | 917K-2 | SH3217 | 3x | Hybrid |
| 246 | C13 | 27914S-11 | 1438K-1 | cv. Rose | 3x | Hybrid |
| 247 | C13 | 27914S-18 | 1438K-1 | cv. Rose | 3x | Hybrid |
| 248 | C13 | 27914S-21 | 1438K-1 | cv. Rose | 3x | Hybrid |
| 249 | C13 | 27914S-22 | 1438K-1 | cv. Rose | 3x | Hybrid |
| 250 | C13 | 27914S-6 | 1438K-1 | cv. Rose | 3x | Hybrid |
| 251 | C13 | 27914S-7 | 1438K-1 | cv. Rose | 3x | Hybrid |
| 252 | C13 | 27914S-8 | 1438K-1 | cv. Rose | 3x | Hybrid |
| 253 | C27 | 27873S-12 | 660K-1 | *malaccensis* 250 | 3x | Hybrid |
| 254 | C27 | 27873S-14 | 660K-1 | *malaccensis* 250 | 3x | Hybrid |
| 255 | C27 | 27873S-17 | 660K-1 | *malaccensis* 250 | 3x | Hybrid |
| 256 | C27 | 27873S-33 | 660K-1 | *malaccensis* 250 | 3x | Hybrid |
| 257 | C27 | 27873S-37 | 660K-1 | *malaccensis* 250 | 3x | Hybrid |
| 258 | C27 | 27873S-7 | 660K-1 | *malaccensis* 250 | 3x | Hybrid |
| 259 | C11 | 26224S-3 | 1201K-1 | SH3362 | 3x | Hybrid |
| 260 | C11 | 26840S-9 | 1201K-1 | SH3362 | 3x | Hybrid |
| 261 | C11 | 26316S-14 | 1201K-1 | SH3362 | 3x | Hybrid |
| 262 | C11 | 26224S-2 | 1201K-1 | SH3362 | 3x | Hybrid |
| 263 | C11 | 26840S-5 | 1201K-1 | SH3362 | 3x | Hybrid |
| 264 | C09 | 25653S-3 | 1201K-1 | SH3142 | 3x | Hybrid |
| 265 | C09 | 26315S-3 | 1201K-1 | SH3142 | 3x | Hybrid |
| 266 | C06 | 28528S-1 | 1201K-1 | Kokopo | 3x | Hybrid |
| 267 | C15 | 26369S-8 | 1438K-1 | Long Tavoy | 3x | Hybrid |
| 268 | C19 | 26530S-1 | 1438K-1 | SH3362 | 3x | Hybrid |
| 269 | C16 | 27528S-1 | 1438K-1 | *malaccensis* 250 | 3x | Hybrid |
| 270 | C16 | 27915S-3 | 1438K-1 | *malaccensis* 250 | 3x | Hybrid |
| 271 | C16 | 28561S-5 | 1438K-1 | *malaccensis* 250 | 3x | Hybrid |
| 272 | C16 | 27915S-2 | 1438K-1 | *malaccensis* 250 | 3x | Hybrid |
| 273 | C16 | 28974S-11 | 1438K-1 | *malaccensis* 250 | 3x | Hybrid |
| 274 | C16 | 28974S-15 | 1438K-1 | *malaccensis* 250 | 3x | Hybrid |
| 275 | C16 | 28974S-22 | 1438K-1 | *malaccensis* 250 | 3x | Hybrid |
| 276 | C16 | 28974S-29 | 1438K-1 | *malaccensis* 250 | 3x | Hybrid |
| 277 | C23 | 29114S-1 | 5610S-1 | *malaccensis* 250 | 2x | Hybrid |
| 278 | C23 | 29114S-14 | 5610S-1 | *malaccensis* 250 | 3x | Hybrid |
| 279 | C23 | 29114S-19 | 5610S-1 | *malaccensis* 250 | 3x | Hybrid |
| 280 | C23 | 29114S-24 | 5610S-1 | *malaccensis* 250 | 3x | Hybrid |
| 281 | C27 | 27873S-26 | 660K-1 | *malaccensis* 250 | 3x | Hybrid |
| 282 | C27 | 27873S-31 | 660K-1 | *malaccensis* 250 | 3x | Hybrid |
| 283 | C27 | 29165S-5 | 660K-1 | *malaccensis* 250 | 3x | Hybrid |
| 284 | C40 | 28506S-1 | Entukura | Calcutta 4 | 3x | Hybrid |
| 285 | C47 | 29364S-2 | Namwezi | cv. Rose | 4x | Hybrid |
| 286 | C50 | 28077S-5 | Nfuuka | 8075-7 | 3x | Hybrid |
| 287 | C05 | 28164S-15 | 1201K-1 | cv. Rose | 3x | Hybrid |
| 288 | C05 | 29285S-20 | 1201K-1 | cv. Rose | 3x | Hybrid |
| 289 | C10 | 26337S-32 | 1201K-1 | SH3217 | 3x | Hybrid |
| 290 | C11 | 27684S-5 | 1201K-1 | SH3362 | 3x | Hybrid |
| 291 | C12 | 24948S-12 | 1438K-1 | 5610S-1 | 3x | Hybrid |
| 292 | C12 | 24948S-21 | 1438K-1 | 5610S-1 | 3x | Hybrid |
| 293 | C12 | 24948S-27 | 1438K-1 | 5610S-1 | 3x | Hybrid |
| 294 | C12 | 29586S-4 | 1438K-1 | 5610S-1 | 3x | Hybrid |
| 295 | C12 | 24948S-22 | 1438K-1 | 5610S-1 | 3x | Hybrid |
| 296 | C12 | 24948S-2 | 1438K-1 | 5610S-1 | 3x | Hybrid |
| 297 | C12 | 24948S-29 | 1438K-1 | 5610S-1 | 3x | Hybrid |
| 298 | C29 | 26820S-1 | 917K-2 | 1968-2 | 3x | Hybrid |
| 299 | C32 | 25474S-5 | 917K-2 | 861S-1 | 3x | Hybrid |
| 300 | C61 | 25974S-18 | 917K-2 | SH3362 | 3x | Hybrid |
| 301 | C61 | 28476S-8 | 917K-2 | SH3362 | 3x | Hybrid |
| 302 | C61 | 25974S-31 | 917K-2 | SH3362 | 3x | Hybrid |
| 303 | C42 | 29275S-1 | Enzirabahima | *malaccensis* 250 | 4x | Hybrid |
| 304 | C42 | 29275S-4 | Enzirabahima | *malaccensis* 250 | 4x | Hybrid |
| 305 | C42 | 29275S-5 | Enzirabahima | *malaccensis* 250 | 4x | Hybrid |
| 306 | C55 | 29636S-1 | Tereza | 7197-2 | 4x | Hybrid |
| 307 | C56 | 28776S-2 | Tereza | 8075-7 | 3x | Hybrid |

2x = diploid, 3x = triploid and 4x = tetraploid
