# Supplementary material for: Trait variation and genetic diversity in a banana genomic selection training population
Source: PLoS One. 2017 Jun 6;12(6):e0178734. doi: 10.1371/journal.pone.0178734 (PMC5460855; doi:10.1371/journal.pone.0178734)
Supplement: S3 Table — (DOCX) [file pone.0178734.s004.docx]

**S3 Table: Summary of all trait variations in response to cycle and field management.**

| Dep. variable | Indep. variable | Sum Sq | Df | F value | Pr(>F) |
| --- | --- | --- | --- | --- | --- |
| NSLF | Clone | 3901.23 | 306 | 3.63 | <0.0001 |
|  | Field | 4.67 | 1 | 1.33 | 0.2492 |
|  | Clone:Field | 1360.46 | 284 | 1.36 | 0.0001 |
|  | Cycle | 2.63 | 1 | 0.69 | 0.4052 |
|  | Clone:Cycle | 1174.64 | 299 | 1.04 | 0.3283 |
| YLSF | Clone | 4790.39 | 306 | 4.50 | <0.0001 |
|  | Field | 2.63 | 1 | 0.75 | 0.3852 |
|  | Clone:Field | 1483.14 | 284 | 1.50 | <0.0001 |
|  | Cycle | 0.00 | 1 | 0.00 | 1.0000 |
|  | Clone:Cycle | 1102.33 | 299 | 0.85 | 0.9669 |
| PHF | Clone | 2222889.11 | 306 | 3.77 | <0.0001 |
|  | Field | 1126.34 | 1 | 0.58 | 0.4449 |
|  | Clone:Field | 432297.46 | 284 | 0.79 | 0.9947 |
|  | Cycle | 8714.88 | 1 | 8.25 | 0.0041 |
|  | Clone:Cycle | 332846.71 | 299 | 1.05 | 0.2662 |
| PG | Clone | 73176.82 | 306 | 4.30 | <0.0001 |
|  | Field | 1.52 | 1 | 0.03 | 0.8686 |
|  | Clone:Field | 12061.30 | 284 | 0.76 | 0.9981 |
|  | Cycle | 351.48 | 1 | 12.11 | 0.0005 |
|  | Clone:Cycle | 13057.24 | 299 | 1.51 | <0.0001 |
| HTSF | Clone | 2151815.75 | 306 | 2.96 | <0.0001 |
|  | Field | 1075.15 | 1 | 0.45 | 0.5014 |
|  | Clone:Field | 895154.77 | 284 | 1.33 | 0.0005 |
|  | Cycle | 59.52 | 1 | 0.02 | 0.8836 |
|  | Clone:Cycle | 976295.15 | 299 | 1.18 | 0.0276 |
| INSL | Clone | 116602.02 | 306 | 2.44 | <0.0001 |
|  | Field | 4.96 | 1 | 0.03 | 0.8584 |
|  | Clone:Field | 58583.77 | 284 | 1.32 | 0.0005 |
|  | Cycle | 141.37 | 1 | 0.79 | 0.3740 |
|  | Clone:Cycle | 51026.49 | 299 | 0.95 | 0.6947 |
| TS^sqrt^ | Clone | 240.28 | 305 | 3.21 | <0.0001 |
|  | Field | 0.24 | 1 | 0.99 | 0.3204 |
|  | Clone:Field | 100.88 | 282 | 1.46 | <0.0001 |
| NSLH | Clone | 4746.65 | 303 | 5.14 | <0.0001 |
|  | Field | 7.50 | 1 | 2.46 | 0.1170 |
|  | Clone:Field | 958.14 | 269 | 1.17 | 0.0417 |
|  | Cycle | 20.74 | 1 | 6.78 | 0.0093 |
|  | Clone:Cycle | 1154.94 | 276 | 1.37 | 0.0002 |
| YLSH | Clone | 2261.86 | 303 | 4.18 | <0.0001 |
|  | Field | 3.33 | 1 | 1.87 | 0.1719 |
|  | Clone:Field | 649.25 | 269 | 1.35 | 0.0003 |
|  | Cycle | 4.14 | 1 | 2.01 | 0.1562 |
|  | Clone:Cycle | 579.70 | 276 | 1.02 | 0.4063 |
| HTSH | Clone | 2714448.28 | 303 | 4.55 | <0.0001 |
|  | Field | 7053.33 | 1 | 3.58 | 0.0587 |
|  | Clone:Field | 1190067.21 | 269 | 2.25 | <0.0001 |
|  | Cycle | 1920.12 | 1 | 0.65 | 0.4196 |
|  | Clone:Cycle | 949051.52 | 276 | 1.17 | 0.0408 |
| BWT^sqrt^ | Clone | 1213.89 | 303 | 12.55 | <0.0001 |
|  | Field | 1.4 | 1 | 4.38 | 0.0365 |
|  | Clone:Field | 126.77 | 269 | 1.48 | <0.0001 |
|  | Cycle | 4.04 | 1 | 15.24 | <0.0001 |
|  | Clone:Cycle | 108.68 | 276 | 1.49 | <0.0001 |
| NH | Clone | 3334.02 | 303 | 8.67 | <0.0001 |
|  | Field | 0.03 | 1 | 0.03 | 0.8713 |
|  | Clone:Field | 569.58 | 269 | 1.67 | <0.0001 |
|  | Cycle | 7.43 | 1 | 6.01 | 0.0143 |
|  | Clone:Cycle | 429.09 | 276 | 1.26 | 0.0048 |
| NF | Clone | 1380508.67 | 303 | 5.46 | <0.0001 |
|  | Field | 112.13 | 1 | 0.13 | 0.7139 |
|  | Clone:Field | 333080.59 | 269 | 1.49 | <0.0001 |
|  | Cycle | 4742.88 | 1 | 6.13 | 0.0134 |
|  | Clone:Cycle | 262980.73 | 276 | 1.23 | 0.0092 |
| FL | Clone | 16284.98 | 300 | 13.49 | <0.0001 |
|  | Field | 33.92 | 1 | 8.43 | 0.0037 |
|  | Clone:Field | 1982.34 | 269 | 1.83 | <0.0001 |
|  | Cycle | 5.95 | 1 | 1.10 | 0.2944 |
|  | Clone:Cycle | 1328.62 | 273 | 0.90 | 0.8661 |
| FC | Clone | 9506.06 | 300 | 16.11 | 0.0000 |
|  | Field | 17.79 | 1 | 9.04 | 0.0027 |
|  | Clone:Field | 733.66 | 269 | 1.39 | 0.0001 |
|  | Cycle | 2.78 | 1 | 1.30 | 0.2548 |
|  | Clone:Cycle | 751.00 | 272 | 1.29 | 0.0021 |
| FRD | Clone | 1003.46 | 299 | 17.55 | 0.0000 |
|  | Field | 2.52 | 1 | 13.19 | 0.0003 |
|  | Clone:Field | 139.73 | 269 | 2.72 | <0.0001 |
|  | Cycle | 0.44 | 1 | 1.75 | 0.1866 |
|  | Clone:Cycle | 70.74 | 271 | 1.04 | 0.3331 |
| PLD | Clone | 865.42 | 299 | 17.60 | 0.0000 |
|  | Field | 2.70 | 1 | 16.42 | <0.0001 |
|  | Clone:Field | 68.27 | 269 | 1.54 | <0.0001 |
|  | Cycle | 0.52 | 1 | 3.03 | 0.0820 |
|  | Clone:Cycle | 60.55 | 271 | 1.29 | 0.0022 |
| PED | Clone | 20.96 | 299 | 11.41 | <0.0001 |
|  | Field | 0.00 | 1 | 0.08 | 0.7799 |
|  | Clone:Field | 16.61 | 269 | 10.05 | <0.0001 |
|  | Cycle | 0.00 | 1 | 0.13 | 0.7192 |
|  | Clone:Cycle | 3.15 | 271 | 0.80 | 0.9913 |

^sqrt^ Original data transformed by square root,
